# Identification and characterization of *GLOBE*, a major gene controlling fruit shape and impacting fruit size and marketability in tomato

**DOI:** 10.1038/s41438-021-00574-3

**Published:** 2021-06-01

**Authors:** Edgar Sierra-Orozco, Reza Shekasteband, Eudald Illa-Berenguer, Ashley Snouffer, Esther van der Knaap, Tong Geon Lee, Samuel F. Hutton

**Affiliations:** 1 University of Florida, Gulf Coast Research and Education Center, 14625 County Road 672, Wimauma, FL 33598 USA; 2 North Carolina State University, Department of Horticultural Science, Mountain Horticultural Crops Research and Extension Center, 455 Research Drive, Mills River, NC 28759 USA; 3 University of Georgia, Center for Applied Genetic Technologies, 111 Riverbend Road, Athens, GA 30602 USA; 4 University of Georgia, Department of Horticulture, 1111 Plant Sciences Bldg, Athens, GA 30602 USA

**Keywords:** Plant breeding, Genetic markers, Plant genetics, Plant genetics

## Abstract

Within large-fruited germplasm, fruit size is influenced by flat and globe shapes. Whereas flat fruits are smaller and retain better marketability, globe fruits are larger and more prone to cuticle disorders. Commercial hybrids are often developed from crosses between flat and globe shaped parents because flat shape is thought to be dominant and fruit size intermediate. The objectives of this study were to determine the genetic basis of flat/globe fruit shape in large-fruited fresh-market tomato germplasm and to characterize its effects on several fruit traits. Twenty-three advanced single plant selections from the Fla. 8000 × Fla. 8111B cross were selectively genotyped using a genome-wide SNP array, and inclusive composite interval mapping identified a single locus on the upper arm of chromosome 12 associated with shape, which we termed *globe*. A 238-plant F_2_ population and 69 recombinant inbred lines for this region from the same parents delimited *globe* to approximately 392-kilobases. A germplasm survey representing materials from multiple breeding programs demonstrated that the locus explains the flat/globe shape broadly. A single base insertion in an exon of Solyc12g006860, a gene annotated as a brassinosteroid hydroxylase, segregated completely with shape in all populations tested. CRISPR/Cas9 knock out plants confirmed this gene as underlying the *globe* locus*. In silico* analysis of the mutant allele of *GLOBE* among 595 wild and domesticated accessions suggested that the allele arose very late in the domestication process. Fruit measurements in three genetic backgrounds evidenced that *globe* impacts fruit size and several fruit shape attributes, pedicel length/width, and susceptibility of fruit to weather check. The mutant allele of *GLOBE* appears mostly recessive for all traits except fruit size where it acts additively.

## Introduction

Tomato is a major vegetable crop worldwide, valued at more than $95 billion annually^1^. Fresh-market tomato has a production value of more than 1.2 billion dollars in the US, with the state of Florida accounting for more than 36% of that value^2^. Fruit shape and size are important traits in fresh market tomato, influencing consumer preference, packing demands, and market value^3,4^.

Tomato originated in the Andean region of South America^5^ and was domesticated in Central America, possibly in Mexico^6^. The size of cultivated tomato fruit has dramatically increased relative to wild species. Fruit of wild tomatoes are two-locular and weigh a few grams. In contrast, a modern tomato fruit may be multilocular and weigh up to 1 kg^6,7^. There is also tremendous variation in fruit shape: whereas fruit of wild tomatoes are simply round, fruit of cultivated tomatoes can be flat, round, fasciated, elongated, pear-shaped, plum, bell pepper-shaped, etc.^6–8^. It is thought that such variation emerged early in the domestication process by selecting alleles with larger fruits and variable shapes, and that these alleles accumulated over time, giving rise to the present-day tomatoes^6,7^.

As many as 30 QTLs likely explain the overall morphological variation in the fruit of tomato^7^. However, most of the variation is explained by mutations in four genes: *SUN*, *OVATE*, *LC*, and *FAS*^9^. While *SUN* and *OVATE* are involved in fruit elongation, *LC* and *FAS* regulate the number of locules in the fruit, which in turn impacts both fruit shape and size. On the other hand, mutations at two loci, *FW2.2* and *FW3.2*, are implicated in the increase in fruit size to a great extent.

The short arm of chromosome 7 harbors *sun*, where a 24.7 kb insertion is linked to elongated fruit shape^10,11^. It was determined that this segment was copied from chromosome 10 by *Rider*, a *Copia*-like retrotransposon, present at both loci^11^. This rearrangement put *SUN* under the control of a defensin (*DEFL1*) promoter, leading to its increased expression in the fruit^11,12^. This mutation results in increased number of cells in the longitudinal area and reduced in the transversal area of the fruit^12^, thus not impacting fruit weight.

*OVATE* encodes a member of the Ovate Family Proteins (OFP), which are believed to be a class of regulatory genes important for plant development^13^. A SNP in the second exon of the *OVATE* gene leads to a premature stop codon^13^, resulting in fruit that can be pear-shaped, round, elongated, or ellipsoid—depending on the genetic background^9,13,14^. *OVATE* interacts with the *sov1* and *sov2* loci on chromosomes 10 and 11, respectively, the first influencing the degree of pear shape and the second fruit elongation^14,15^.

*FAS* and *LC*, located on chromosomes 2 and 11, respectively, regulate the number of locules in the fruit, which in turn impacts both fruit shape and size^6,16–19^. The *fas* mutation results from a 294-kb inversion that impairs the expression of *SlCLV3*^20^. It is thought that *FAS* impacts meristem organization and boundary formation within the floral meristem, resulting in changes in the number of locules in the fruit^16,21^. On the other hand, *SlWUS* is the gene underlying the *lc* mutant, which controls the inflorescence and floral meristem formation phase ultimately affecting the final size of the fruit^15,19^. Two SNPs 1080 bp downstream of this gene are believed to cause the phenotype by disrupting the regulatory region of this gene, resulting in increased expression^15,19^. Potentially involved in the *SlWUS-SlCLV3* network is *ENO*, a recently identified transcription factor of the AP2/ERF superfamily which may act by regulating *SlWUS* expression^22^. A natural 85-bp deletion in the promoter of *ENO* leads to decreased expression of this gene and increased locule number, and evidence suggests that this mutation occurred prior to tomato domestication and is now fixed in cultivated tomato^22^.

*fw2.2* is responsible for as much as 30% of the difference in fruit size between modern and wild tomatoes^23^. *fw2.2* increases fruit weight by causing the expansion of the placenta and columella of the fruit^15^. *FW2.2* is a cell number regulator (*CNR*) located on the bottom of chromosome 2, and it is believed that a mutation in the promoter region increases fruit size by influencing the rate of cell division^23,24^.

*FW3.2* is located on chromosome 3 and is as a member of the Cytochrome P450 gene family (formerly identified as *SlKLUH*) whose members are known to control organ and plant size^25–27^. The increase in fruit weight is the result of pericarp and septum expansion. *SlKLUH* likely effects these changes by influencing the duration of cell division, thereby increasing the number of cells^15,27^. Although, it was initially believed that a SNP in the promoter region of the *SlKLUH* gene was responsible for *fw3.2*, it was recently discovered that a ~50-Kbp tandem duplication at this locus resulted in two copies of *SlKLUH*, thus affecting fruit size through increased gene expression^27,28^. The expression of *SlKLUH* is exceptionally high in the growing seeds^27^, suggesting that a seed-produced compound may be involved in the increase in fruit mass^27^. Other important fruit size and shape QTLs are *fs8.1*, controlling fruit elongation, and *fw11.3* which explained from 8 to 13% of fruit weight variation in populations between domesticated and wild tomato accessions^29,30^.

Whereas the above-mentioned loci account for major fruit shape and size variation between wild and cultivated germplasm and among some market classes of cultivated tomato, the genetics of shape and size variation within specific market classes is less well understood. A study evaluated the variation for six major fruit shape and size loci (*sun, lc, ovate, fas, fw2.2*, and *fw3.2*) and found that there was no variation among sampled fresh market large-fruited tomato inbred lines from the UF/IFAS and NCSU breeding programs^31^. Specifically, the set of breeding lines genotyped from both programs are fixed for the domesticated allele at *FW2.2, FW3.2, FW11.3*, and *LC*, and fixed for the wild allele at *FAS*, *OVATE*, and *SUN*^31^. Hence variation in shape/size within this market class is explained by another, as yet unknown, locus(loci).

In large-fruited, fresh market tomato, flat and globe fruit shapes are often used by breeders in the development of hybrid cultivars. Flat fruit are wide and ‘blocky’ and are usually smaller than globe fruit, which are deep and round (Fig. S1). Globe fruit shape is described in some cultivar releases as being associated with susceptibility to cuticle disorders, including radial cracking and weather check (cuticle cracking that occurs primarily on fruit shoulders under high moisture conditions, Fig. S1)^32–34^. Hybrids between flat-fruited and globe-fruited parents, however, are thought to be intermediate in fruit size, while retaining the fruit shape and marketability characteristics of the flat parent (Fig. S1). The genetics of flat and globe fruit shape are currently unknown, but identification of the underlying loci may be of particular use for breeders.

The source of globe fruit shape in the tomato breeding program of the University of Florida Institute of Food and Agricultural Sciences (UF/IFAS) is 74VF18, an inbred line from the University of California at Davis (J.W. Scott, personal communication). 74VF18 is likewise in the pedigree of Fla. 7060, the globe parent in the hybrid ‘Solar Set’ and the source of globe fruit shape in the North Carolina State University (NCSU) tomato breeding program (R.A. Gardner, personal communication). Previous observations suggest that flat/globe fruit shape is simply inherited (R.A. Gardner, personal communication). Moreover, globe-fruited germplasm from the UF/IFAS and the NCSU tomato breeding programs has been distributed to many public and private breeding programs over the years, and it is possible that the genetic control of this fruit shape characteristic is common among various tomato breeding programs.

The objectives of this research were: (1) to characterize the genetic basis of flat and globe fruit shapes in large-fruited fresh-market tomato, and to identify and fine-map the underlying locus(loci); (2) as genetic control is determined, to explore the extent to which this locus(loci) may explain fruit shape variation among a diverse panel of germplasm from the UF/IFAS and NCSU public breeding programs, and from several private breeding programs; (3) to identify the gene(s) underlying *globe* using CRISPR/Cas9 knock outs and to inquire its origin in the domestication process; and (4) to quantitatively characterize the locus(loci) for its effect on fruit size, fruit shape, fruit disorders, and other plant traits.

## Results

### Identification of *globe*, a major locus controlling flat and globe fruit shapes

**Fig. 1:**
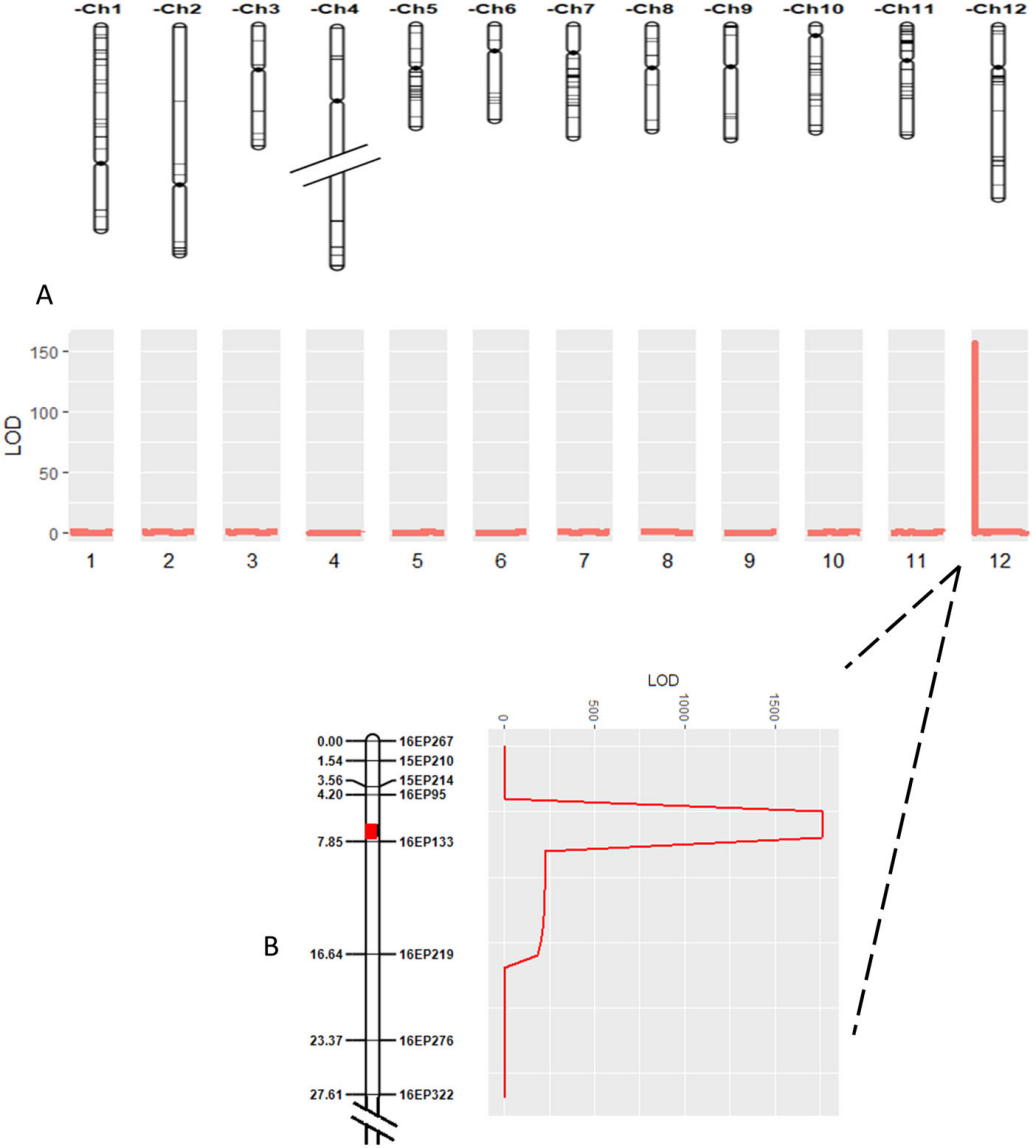
Identification of a major fruit shape QTL on the short arm of chromosome 12. **A** Inclusive composite interval mapping (ICIM) analysis based on 751 markers segregating among 12 globe and 11 flat F_6_ recombinant inbred lines from the Fla. 8000 × Fla. 8111B population. **B** ICIM analysis based on eight markers located within the target interval on chromosome 12 and tested on 238 F_2_ plants from the Fla. 8000 × Fla. 8111B population. Marker names are presented to the right of the chromosome figure, and centi-Morgan positions are provided to the left. The thick red band on the chromosome identifies the QTL position

Phenotyping of 83 F_6_ RILs identified 39 with flat and 44 with globe fruit shape, and a chi-square test for a 1:1 suggested that fruit shape was controlled by a single locus (*X*^2^ = 0.15, *P*-value = 0.7). From this group, 11 flat and 12 globe-shaped RILs were selected and individually genotyped with a 7720 SNP array, identifying 751 polymorphic loci. Inclusive composite interval mapping (ICIM) analysis with this information detected a single locus in the upper arm of chromosome 12 that was significantly associated with fruit shape (Fig. 1A).

A 238-plant F_2_ population, comprised of 176 flat and 62 globe individuals provided an acceptable fit to the phenotypic ratio 3:1, supporting a single locus (*X*^2^ = 0.07, *P*-value = 0.79) and indicating dominant gene action by the flat shape. ICIM analysis on this population, using eight new sequence-based markers spanning the chromosome 12 interval from 390,069 to 2,877,860 bp confirmed the presence of a major QTL explaining 99.58% of the phenotypic variation for fruit shape in this region (Fig. 1B; Table S1). This locus will hereafter be termed the *globe* locus.

### Fine mapping of the *globe* locus

**Fig. 2:**
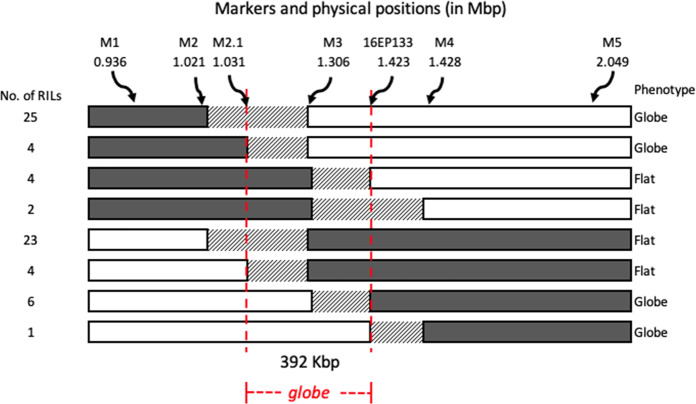
Fine mapping of the *globe* locus based on genotypes and phenotypes of 69 recombinant inbred lines (RILs). Results delimited the locus to an approximately 392 Kbp region (delimited by the vertical red dashed lines). The physical position of the markers is based on the Tomato Genome Build SL4.0. Each distinctive recombinant genotype is represented by a horizontal bar, and the number of RILs with this genotype is indicated to the left. Shading of the bars represents the two haplotypes of each recombinant group: dark gray, homozygous for the Fla. 8000 (flat) allele; white, homozygous for the Fla. 8111B (globe) allele; diagonal stripes, undetermined

F_3_ and F_4_ segregating populations were screened with seven markers saturating the region between 936,308 and 2,049,214 bp, and 69 recombinant plants were identified. From these, RILs for fine mapping RILs (FM-RILs) were developed, phenotyped for fruit shape, and genotyped with the seven markers. Genotypes and phenotypes of the 69 FM-RILs are presented in Fig. 2. The results clearly delimited the *globe* locus to an approximately 392 Kbp region between 1,031,490 (marker M2.1) and 1,423,513 bp (marker 16EP133). Marker M3 demonstrated complete co-segregation with the phenotype throughout the entire FM-RIL population.

### Validation of the *globe* locus

Results were validated using a separate F_2_ population from the cross between Fla. 7946 (flat) and Fla. 8442 (globe). One hundred and three plants were phenotyped for fruit shape and genotyped with the M3 marker, among which, 81 were flat, and 22 were globe, fitting a 3:1 ratio and supporting a single dominant locus (*X*^2^ = 0.38, *P*-value = 0.54). All plants with globe fruit shape were homozygous for the globe allele at marker M3; and among the plants with flat fruit shape, 20 were homozygous for the flat allele, and 61 were heterozygous. These genotypes also fit a 1:2:1 ratio (*X*^2^ = 1.82, *P*-value = 0.40), indicating no distortion in Mendelian segregation.

To investigate the efficacy of this QTL across backgrounds, fruit shape phenotype and genotype at the *globe* locus were determined for three globe-shaped inbred lines (Fla. 8735, Fla. 7776, and Fla. 8022), for three flat near isogenic lines (NILs) that corresponded to each globe inbred, and for F_1_s resulting from the cross between each globe recurrent parent and its flat NIL. Each NIL was developed by a modified backcrossing approach, without the use of molecular markers, to introgress the flat fruit shape phenotype into the recurrent parent. Notably, whereas each of the globe parents were homozygous for the globe allele, all three of the flat NILs were homozygous for the flat allele, and each F_1_ was heterozygous and had flat fruit shape. These results further support the ability of the *globe* locus in explaining variation of flat and globe fruit shape in multiple backgrounds.

### Distribution of *globe* among tomato breeding programs

To explore the extent to which the *globe* locus explains fruit shape variation across a broader set of germplasm, a panel of 204 inbred lines from multiple breeding programs were genotyped and surveyed for fruit shape. Of 176 large-fruited samples surveyed, 129 had flat fruit shape, and 47 had globe fruit shape (Table S4). Each of the inbred lines were genotyped with six markers located on chromosome 12 between 0.971 and 1.895 Mbp (Table S1). Results identified nine different marker haplotypes among 129 flat-fruited lines (Table S4). Haplotypes of the flat inbred lines differed for genotype at all markers except M3, where all inbreds had the flat allele. On the other hand, five different haplotypes were identified among globe inbred lines. As with the flat inbred lines, haplotypes of the globe lines differed for genotype at all markers except M3, where each showed the globe genotype. Thus, genotype at marker M3 consistently corresponded to fruit shape phenotype for all 176 of the large-fruited inbred lines that were surveyed. Five of the marker haplotypes observed among large-fruited inbred lines were also found in cherry, grape, and plum types. Each of the five grape/cherry inbred lines were homozygous for the flat allele at marker M3. However, genotype at this marker was not consistent among the 22 plum lines surveyed, where the flat and globe alleles were equally frequent.

To ascertain the extent to which large-fruited commercial hybrids involve crossings between flat and globe parents, 35 hybrids were surveyed with marker M3 (Table S5). From those hybrids, 17 were homozygous for the flat allele, and 18 were heterozygous. None of the hybrids surveyed were homozygous for the globe allele. These results suggest that globe-fruited inbred lines are commonly used as parents of commercial hybrids, but hybrids are being developed from crosses between two flat parents with equal frequency.

### Candidate gene identification, confirmation of the *GLOBE* gene, and insights into its origins during the domestication process

#### Solyc12g006860 identified as the only candidate gene

**Fig. 3:**
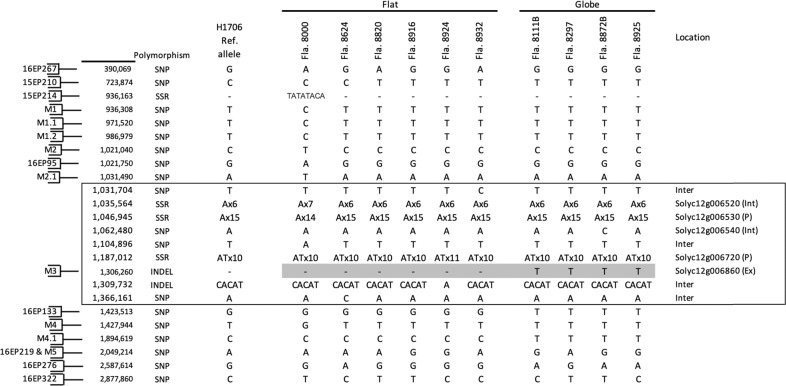
Chromosome 12 sequence polymorphisms identified from comparison of whole-genome resequencing alignments for 6 flat and 4 globe UF/IFAS breeding lines. Select polymorphisms that were used for marker development, and all polymorphisms within the 392 Kbp fine mapped interval (indicated by the box) for the *globe* locus are displayed. Physical positions (bp) are displayed along the left of the figure and correspond to version SL4.0 of the Heinz 1706 genome assembly. Loci corresponding to markers used for mapping and fine mapping are indicated by brackets. Location of polymorphisms relative to annotated genes are displayed to the right and are indicated by: P = within the promoter region, corresponding to approximately 1.0 Kbp upstream of the annotated gene; Int = within an intron of the annotated gene; Ex = within an exon of the annotated gene; Inter = located intergenically

Whole-genome sequencing alignments for 6 flat and 4 globe inbred lines were investigated for sequence polymorphisms occurring in the 392 Kbp fine-mapped interval at the *globe* locus. Comparative analysis identified nine polymorphisms between markers M2.1 and 16EP133: 4 SNPs, 3 simple sequence repeats (SSR), and 2 INDELs (Fig. 3). Eight of those polymorphisms were not consistently polymorphic between flat and globe lines and showed genotypic variation within either group. However, the indel corresponding to marker M3, which demonstrated complete co-segregation with fruit shape in all FM-RILs and consistently corresponded with fruit shape in the germplasm survey, was also consistently polymorphic between the flat and the globe lines that were sequenced.

Fifty annotated genes are located within the 392 Kbp fine mapped interval (ITAG4.1, Table S6). Among the polymorphisms identified in this region, four are intergenically located, two are located in an intron of genes Solyc12g006520 and Solyc12g006540, and two are located within the promoter region of genes Solyc12g006530 and Solyc12g006720. However, none of these consistently corresponded to fruit shape among the sequenced lines. Alternatively, the indel at position 1,306,260, which corresponds to marker M3, is located within the final exon of Solyc12g006860, a gene annotated as a brassinosteroid hydroxylase (Fig. 3). The coding sequences of the wild-type and mutant alleles of Solyc12g006860 were translated into amino acid sequences and aligned for comparison (Fig. S2). Whereas the predicted protein of the flat allele is 555 amino acids in length, the single base pair insertion in the globe allele causes a frameshift mutation, resulting in 16 amino acid substitutions followed by a premature stop codon which truncates the protein by eight amino acids.

Because no other mutations were found in the coding regions of any predicted genes in this area, and the indel at this gene is the only polymorphism demonstrating complete segregation with shape, these results collectively identified Solyc12g006860 as the most likely candidate gene underlying the flat/globe locus.

**Fig. 4:**
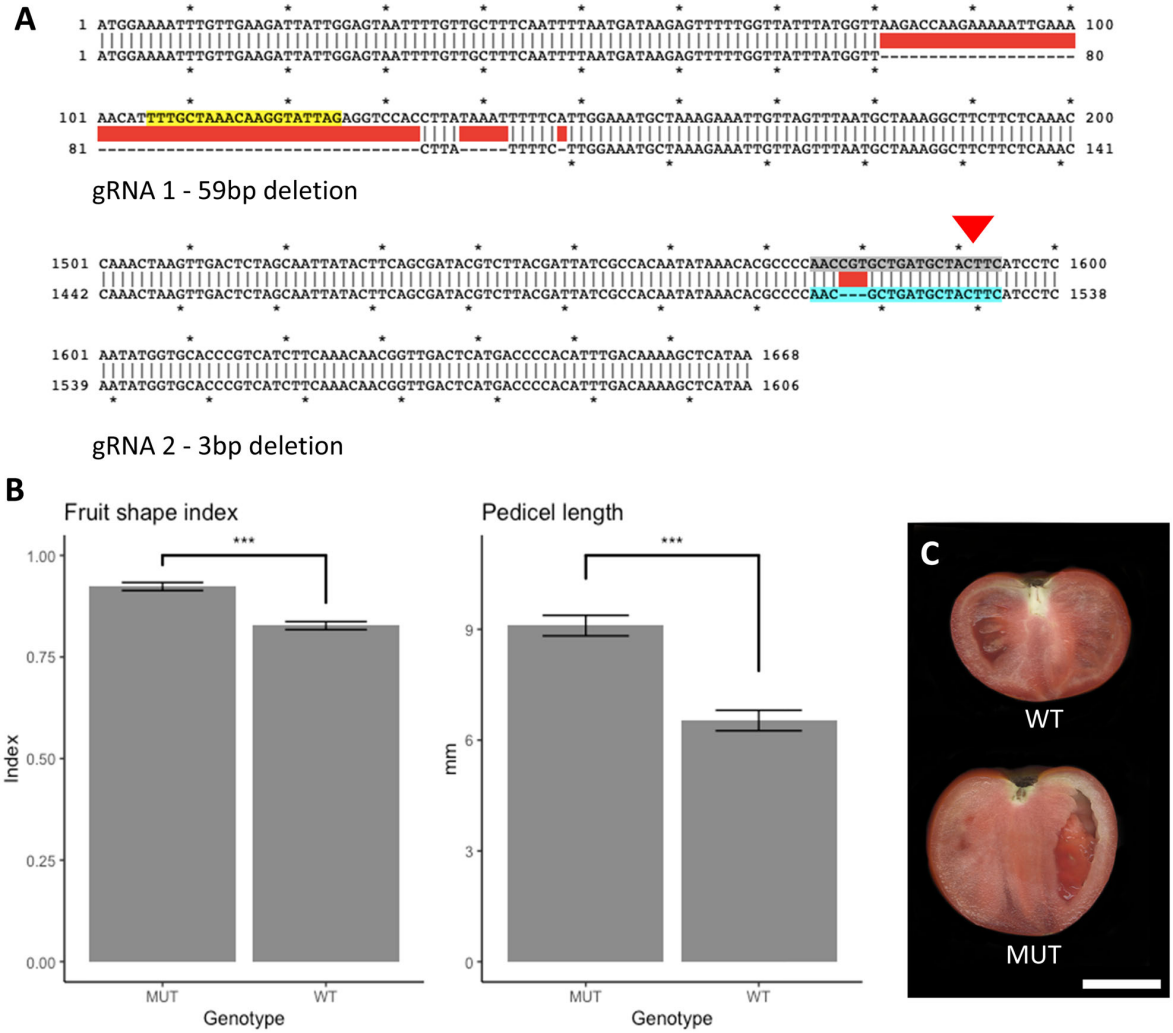
CRISPR/Cas9 knock out confirms Solyc12g006860 as the *GLOBE* gene. **A** cDNA sequence of Solyc12g006860 showing the two guide RNAs (1, yellow shade; and 2, gray shade) and the two deletions caused in the gene (the first of 59 bp, the second of 3 bp). The inverted red triangle shows the location of the natural *globe* mutation (insertion of a thymine). **B** Phenotypic measurements of fruit shape index and pedicel length in Fla. 8059 (WT) and CRISPR/Cas9 knocked out plants (MUT). MUT and WT are significantly different with *P* < 0.001, error bars represent the standard error of the mean (*n* = 5 plants; each observation was derived from the average of at least 3 fruits). **C** Sliced fruit of each of the wild-type (upper) and mutant plants (lower). Scale bar is 2.5 cm

To confirm Solyc12g006860 as the *GLOBE* gene, CRISPR/Cas9 targeted mutagenesis was employed to knock out the gene in Fla. 8059, a flat-shaped line. A single mutation event was identified, containing two deletions within the gene: a 59 bp deletion at the beginning of the coding sequence, and a 3 bp deletion towards the end of the gene (Fig. 4A). Both mutations were present in the mutant plants measured. A T0 plant was selfed to generate homozygous mutants that remained Cas9 positive, and fruit shape and pedicel length were measured in the T1 generation. As expected, plants mutant for Solyc12g006860 had a higher fruit shape index and increased pedicel length compared to wild-type plants (Fig. 4B, C), resembling the phenotype of the globe-shaped lines (further discussed in the phenotypic characterization section). These results confirmed Solyc12g006860 as *GLOBE* and support that the single base pair insertion in the last exon of the natural mutant results in a gene knock out, thereby producing the globe phenotype.

#### Insights into the origin of globe during domestication

The presence of the mutant allele of *GLOBE* in a broad set of germplasm was assessed *in silico* using publicly available data. The sequencing data provided genotypic information on a total of 595 unique accessions, including tomato wild relative species, semidomesticated cultivars (*S. lycopersicum* L. var. *cerasiforme*) and fully domesticated tomato varieties (*S. lycopersicum* L. var. *lycopersicum*). Among these accessions, only three cultivated accessions harbored the mutant allele: Fla. 7060, TS-103 (EA00389), and TS-306 (Supplementary file 2). Fla. 7060 is a globe-shaped breeding line from the UF/IFAS breeding program, which was homozygous for mutant allele as expected. TS-103 (EA00389) was heterozygous and TS-306 appeared homozygous (based on a single read at this position). No ancestral accessions contained the mutant allele, suggesting that the mutation arose very recently during the domestication process.

### Phenotypic characterization

#### Fruit size

**Fig. 5:**
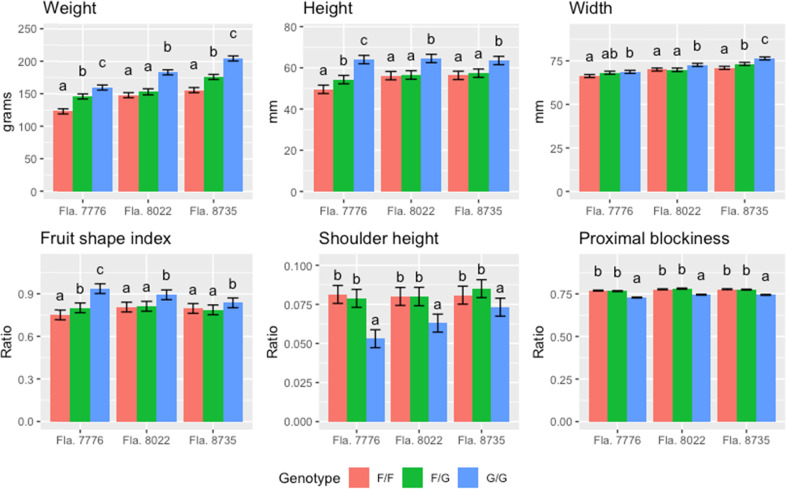
Average values for six fruit size and shape attributes measured across three seasons of trialing for each of three backgrounds and presented according to the genotype at the *globe* locus (F/F, homozygous flat; F/G, heterozygous; G/G, homozygous globe). The bars represent the standard error of the mean. Within each background, different letters represent significantly different means (Tukey HSD test, alpha = 0.05, based on a 4-replicate RCBD, trialed three seasons [*N* = 12]). The response value of each experimental unit comes from: the average fruit weight from four or five harvests each season [yield/total fruit per plot] (weight), or average values of 24 fruits per experimental unit from second to fourth harvest (fruit shape attributes)

The impacts of flat and globe fruit shape were evaluated in three near isogenic backgrounds. The analysis indicated that there were significant differences in fruit weight between genotypes, backgrounds, and their interaction. The average fruit weight of globe genotypes was consistently greater than that of the flat genotypes in all backgrounds, and the heterozygote fruit weight was intermediate between both homozygotes for two of the backgrounds, indicating a possible additive effect for this trait (Fig. 5). The overall mean fruit weight was 143 (±2.60) for flat, 159 (±2.76) for heterozygous, and 183 (±2.60) g for globe genotypes, indicating an increase in weight of approximately 11% in heterozygous and 27% in globe genotypes relative to the flat genotype. Likewise, yields of extra-large fruit were generally highest for globe and heterozygous genotypes, and yields of large fruit were generally highest for flat and heterozygous genotypes (Table S7).

#### Marketability of the fruit

Marketable yield results showed high variation between the spring 2018 and both fall seasons. In fall seasons, whereas the heterozygous genotypes had higher yield compared to the homozygous flat genotypes (although not always significantly different), the homozygous globe genotype had the highest yield in two of the three backgrounds. On the other hand, in spring 2018, homozygous globe genotypes consistently had the lowest marketable yield (Table S7).

With respect to culls, fall 2017 results indicated no significant differences among genotypes except for the Fla. 7776 background, where the homozygous flat genotypes had the highest percentage of culls. In contrast, the homozygous globe genotype had the highest percentage of culls in all backgrounds in spring 2018 and fall 2018. These differences were significant in all backgrounds except for Fla. 8022 in spring 2018 (Table S7).

The two fruit disorders that contributed the most to culls were radial cracking and weather check. With respect to radial cracking, there were no significant differences among genotypes in any background in fall 2017. In spring and fall 2018, the homozygous globe genotype typically had the highest percentage of radial cracking, and the heterozygous genotype tended to have the smallest percentage or was not significantly different from the homozygous flat genotype (Table S7). With respect to weather check, the homozygous globe genotype in the Fla. 7776 background demonstrated the highest percentage of weather check across all seasons, having more than six times the culls as the other two genotypes. For the other two backgrounds, results were not consistent in the fall seasons, but in spring 2018, the homozygous globe genotype had the highest percentage of culls among backgrounds (albeit the difference was not significant in the Fla. 8022 background, Table S7).

The relationship between weather (rain and temperature) and percentage of fruit with weather check damage was assessed by collecting weather data for each season and plotting it against the percentage of culled fruit with weather check damage by genotype (Fig. S3). Fruit with weather check damage appeared a few days after a rain events were recorded; furthermore, the percentage of damaged fruit appeared to be correlated to the amount of rainfall. Notably, in spring 2018, rainfall was frequent, and the proportion of damaged fruit was the highest of all three seasons. Moreover, the homozygous globe genotypes were by far the most affected. On the other hand, in both fall seasons, where the rain was less frequent, the percentage of damaged fruit was lower, yet still highest for the globe genotypes. These results support the significance of rainfall in the susceptibility of fruit to weather check damage, especially in globe genotypes.

#### Fruit shape

All basic shape measurements (fruit height, width, area, and perimeter; data not shown for perimeter) were significantly affected by genotype at the *globe* locus, with the homozygous globe genotype consistently having the highest values, and the homozygous flat genotype having the lowest (Figs. 5 and S4). The heterozygous genotype was intermediate (though not always significantly different) to the homozygous genotypes. Additionally, the differences in fruit width among genotypes were relatively small compared to those observed for fruit height, indicating that changes in fruit weight are primarily due to differences along the longitudinal axis of the fruit.

The analysis of the fruit shape index (FSI) resulted in significant differences among genotypes and interaction between the background and the genotype (Fig. 5). Still, the homozygous globe genotype consistently had the highest FSI across all backgrounds. On the other hand, the FSI of the heterozygous genotype was not significantly different from the homozygous flat genotype in two of the three backgrounds (in the Fla. 7776 background it appeared intermediate).

The *globe* locus significantly affected fruit shape at the proximal (stem) end of fruit. Although the analysis suggested a significant interaction between genotype and background for all proximal end traits (shoulders, proximal end angles, and proximal indentation area, Figs. 5 and S4), the homozygous globe genotype always resulted in significantly smaller values, and the Fla. 8022 and Fla. 8735 backgrounds resulted in similar values for both homozygous flat and heterozygous genotypes. This is in agreement with the less prominent shoulders observed in globe fruits. On the other hand, in the Fla. 7776 background, the heterozygous genotype appeared significantly different and intermediate between both homozygotes for all traits except for shoulder height. Contrary to the proximal end, the locus did not appear to have a large impact in the distal end of the fruit, where only the Fla. 7776 background had significant and consistent differences among genotypes for distal angle (Fig. S4).

According to the blockiness descriptors (proximal, distal, and fruit shape triangle Figs. 5 and S4), the proximal width of the fruit appeared to be more affected compared to the distal width. Notably, the analysis of the proximal blockiness and fruit shape triangle resulted in a non-significant genotype by background interaction, with the homozygous globe genotypes consistently having the lowest ratios for both descriptors, and the heterozygous genotype values similar to those of the homozygous flat genotype. On the other hand, the distal blockiness was significantly affected by the genotype only in two of the backgrounds, and the differences between genotypes appeared smaller (Fig. S4). This further supports the lower impact of the locus on the distal end, compared to the proximal.

The greater impact of the locus on the proximal blockiness was also evidenced by the asymmetry descriptors. While no consistent differences were found for the vertical asymmetry, the horizontal asymmetry and the ovate descriptors resulted in significant differences among genotypes with no significant interaction with background (Fig. S4, data not shown for vertical asymmetry). The homozygous flat and heterozygous genotypes were consistently similar and significantly different from the homozygous globe genotype across all backgrounds, with globes being more symmetric and less top-heavy.

The difference in shape between the globe genotype and the flat and heterozygous genotypes was also demonstrated by the elliptic and circular fit functions which yielded very similar results. For these descriptors, the globe genotype had the highest *R*^2^ across backgrounds. The heterozygous genotype appeared comparable to the homozygous flat in all backgrounds except for the Fla. 7776, where it appeared intermediate between the homozygous genotypes (Fig. S4, data shown only for elliptic fit).

#### Effects on plant biomass and fruit pedicel

The plant biomass of the homozygous globe genotype was significantly lower in two of the three backgrounds (Fla. 7776 and Fla. 8735), compared to the heterozygous and homozygous flat genotypes. In those two backgrounds, the plant biomass was reduced by 12–16% in the globe genotypes compared to the flat. Although the mean of the globe genotype was the lowest in the Fla. 8022 background, the analysis showed no significant differences among genotypes in this background (Table S8).

Fruit pedicel size was significantly different among genotypes. For pedicel length, although the analysis suggested that the interaction between genotypes and backgrounds was significant, the pedicel was consistently longest in the homozygous globe genotype (~19 mm), and intermediate in the heterozygous (~10–12 mm) but closer in length to the homozygous flat genotype (~9–11 mm). Similarly, the homozygous globe genotype consistently had the thinnest pedicel among backgrounds (~3.3 mm). The pedicels of the homozygous flat and heterozygous genotypes had similar values in all backgrounds (~4–4.5 mm) except for the Fla. 7776 background, where the heterozygous was significantly different from both homozygous genotypes (Table S8).

## Discussion

Previous studies have identified many major and minor QTLs controlling fruit size and/or shape. Such studies were mainly carried out by crossing domesticated tomato with wild accessions. Thus, the QTLs represent critical fruit shape/size loci involved in the transition from wild to modern tomato. In contrast, the present study focused on fruit shape and size variation existing within large-fruited cultivated tomato. Evidence suggests that there is no allelic variation at known major fruit shape and size loci (*sun, lc, ovate, fas, fw2.2*, and *fw3.2*) in fresh market large-fruited germplasm from the UF/IFAS breeding program^31^. In fact, the study by Blanca et al.^31^ included the parents of the mapping population (Fla. 8000 and Fla. 8111B) and found that all materials were fixed for the domesticated allele at *FW2*.2, *FW3.2*, *FW11.3* and fixed for the wild-type allele at *SUN*, *FAS*, and *OVATE*, suggesting that other locus(loci) explain variation in fruit shape and size in these materials.

This study identified *GLOBE* as a major fruit shape gene on the upper arm of chromosome 12. This chromosome is not known to harbor any major effect shape or size genes. However, former studies detected fruit shape/size QTLs in this area of chromosome 12. For instance, one review summarized QTLs controlling fruit shape and size that were mapped during the 15 years prior to the study^7^. Seven QTLs affecting fruit shape or size in chromosome 12 were noted, two of which were mapped to the upper arm*: fs12.a* and *fw12.1*. In another study, two QTLs controlling FSI and distal end angle in the upper arm of chromosome 12 were observed in F_2_ and BC_1_ populations from a cross between the elongated fruit shape Howard German cultivar and *S. pimpinellifolium*^35^. Fruit shape QTLs in this region were also detected in an F_2_ population between the elongated-fruit tomato cultivar Sausage and *S. pimpinellifulium*^36^. While different fruit shape traits were found in each study, a common QTL may segregate in all studies. However, as demonstrated by the in-silico germplasm survey, the *globe* mutation likely arose late in domestication, and was not present in any of the ancestral accessions surveyed. Thus, although *fs12.a* and *fw12.1* could relate to the *globe* locus, the QTLs detected in studies involving *S. pimpinellifolium* seem to be distinct.

In our study, the single-base thymine insertion towards the end of the coding sequence of Solyc12g006860 completely co-segregated with fruit shape in all populations tested and among all large-fruited inbred lines surveyed, placing this gene as the only likely candidate. CRISPR/Cas9 mutants confirmed that this gene harbors the *globe* mutation. The allele for flat fruit shape, which is identical to that of the Heinz 1706 reference genome and seemingly corresponds to the wild-type allele, translates into a 555-amino acid peptide. Both wild-type and mutant proteins have the same peptide sequence up to the 531^th^ position. Beyond this point, the mutant allele differs in sequence for 16 consecutive amino acids before truncating prematurely at position 547 (Fig. S2). The CRISPR/Cas9 mutant created in this study had two deletions in Solyc12g006860: one of 59 bp at the beginning of the coding sequence and another of 3 bp towards the end. The mutations knocked out the gene and created the globe phenotype in a flat-shaped breeding line. This evidence confirmed that the *globe* mutation is a loss of function mutation.

Solyc12g006860 is annotated as brassinosteroid hydroxylase (ITAG4.1 gene annotation) and belongs to the Cytochrome P450 family of genes. Brassinosteroids are steroidal hormones distributed ubiquitously in plants and are involved in the regulation of important processes like cell development growth and multiplication^37^. Previously, the expression of Solyc12g006860 was characterized and was determined that it functions in brassinosteroid catabolism^38^. This suggests that altered regulation of brassinosteroids by the different alleles at *GLOBE* may influence cell elongation and division, leading to the change in fruit morphology.

Interestingly, *FW3.2* (*SlKLUH*) is also a member of the Cytochrome P450 gene family. It is presently understood that increased expression of *SlKLUH* originated by the tandem duplication at the *FW3.2* locus is what drives increased fruit mass^28^. In contrast to *SlKLUH*, Solyc12g006860 contains a mutation within the coding region resulting in a loss of function rather than in a change of gene expression.

Although the effect of *SIKLUH* on fruit size is observed in the pericarp of fruit, its expression is particularly high in developing seeds, and it is therefore speculated that *SlKLUH* regulates fruit weight through a seed-produced compound, similar to *KLUH* in *Arabidopsis*^27,39^. Interestingly, a transcriptome mapping study of tomato fruit development and ripening determined that expression of Solyc12g006860 is likewise highest in developing seeds^40^. Besides providing circumstantial evidence supporting Solyc12g006860 as the *GLOBE* gene, these findings suggest that *SlKLUH* and Solyc12g006860 may function similarly in the regulation of fruit size and shape. Further research is needed to determine the physiological basis of *globe* on fruit morphology.

The *globe* mutation provides a useful molecular resource for breeders and geneticists who may desire to distinguish flat and globe genotypes. The survey of commercial hybrids indicates that although globe parents are being used in some crosses, other hybrids are being developed by crossing two flat parents. Hybrid cultivar releases from UF/IFAS and NCSU alike remark on the advantage of intercrossing flat and globe parents to boost fruit size in the hybrid.

In fresh-market tomato, breeders have for many years inter-crossed flat and globe-shaped inbred lines to create commercial hybrids that, in theory, capitalize on the larger size of the globe parent and the fruit quality characteristics of the flat parent. Yet although this trait has been utilized for so many years, the genetics of flat and globe fruit shape was formerly unknown, and its actual impacts on fruit size and quality were not formerly characterized. Our results demonstrated that although *globe* has a relatively additive effect on fruit size, it acts in a recessive manner on many fruit shape attributes. However, this should be interpreted with care, as illustrated by the more additive gene action in the Fla. 7776 background for several fruit shape descriptors. Other genetic factors in this background may modify the effects of the locus on fruit shape. Results also indicate that although the globe fruit shape is more prone to imperfections that reduce marketability (like weather check, which is also dependent on the environment), heterozygosity for the QTL results in similar marketability rates as flat fruit shape. Thus, compared to the flat genotype, the heterozygote has increased size and similar shape and quality, with no penalty in yield. Furthermore, in light of the findings that a large number of current commercial cultivars do not involve crosses between flat and globe parents (Table S5), these data provide further evidence that fruit size could be increased in such hybrids through the introduction of the globe allele into one of the hybrid’s parents.

*globe* had a greater effect on fruit height than on fruit width, and this is likely the primary factor driving changes in fruit size. Furthermore, fruit shape attributes related to the proximal end of the fruit were those primarily affected by this locus. In comparison, *fw3.2* increases fruit weight mainly by increasing cell number in pericarp and septum tissues^27^, and it has a minor effect on shape—primarily affecting the proximal end of the fruit^41^. On the other hand, there are three major QTLs associated with fruit elongation: *sun*, *ovate*, and *fs8*.1 (ref. ^29,36^). *sun* does not affect fruit size because the increase in height is compensated by a reduction in width^12^; *fs8.1* causes an increase in fruit height with no reduction in width^29^; *ovate* results in different elongated shapes depending on the genetic background with a slight reduction in fruit weight^13^. Thus, *globe* has an apparently unique effect, as it impacts both fruit shape and size, and it seems to have a greater proximal end impact than *fw3.2*.

Besides the effect on fruit size, it was found that transgenic lines for *FW3.2* were affected for plant height, leaf size, and seed number, implying a pleiotropic effect on development^27^. *globe* also has an effect on plant biomass and pedicel length, where homozygosity for the mutant allele results in a reduced plant biomass and longer and thinner fruit pedicels. Furthermore, differences in seedling height were observed between the globe breeding lines Fla. 7776, Fla. 8022 and Fla. 8735, and their respective flat NIL and F_1_, where seedlings homozygous for the globe allele were taller (7–34%) and appeared thinner and leggier at 27 days after sowing than either homozygous flat or heterozygous seedlings (24 < *n* < 77, data not shown). Thus, similar to *FW3.2*, *GLOBE* may also have pleiotropic effects on plant growth. More work is necessary to elucidate the effect of this gene on seedling and young plant growth, and cell size and cell number in the fruit.

## Materials and methods

### Plant materials for mapping and fine mapping

Several populations developed from the cross between the breeding lines Fla. 8000 and Fla. 8111B were used for mapping and fine mapping the locus controlling flat and globe fruit shape. Fla. 8000 and Fla. 8111B have flat and globe fruit shape, respectively. Eighty-three F_6_ RILs were grown in the field and phenotyped for fruit shape. From those, 23 RILs (12 with globe fruit shape, 11 with flat fruit shape) were sampled for initial genotyping using the Illumina Infinium SolCAP tomato SNP array (described below). A 238-plant F_2_ population from the same breeding lines was likewise grown in the field and phenotyped for fruit shape for confirmation of the mapping results from the RILs. Fine mapping was conducted in F_4_ and F_5_ populations from the same cross following a map-based approach.

Validation of the identified locus was conducted using additional populations. These included a 103-plant F_2_ population from the cross between Fla. 7946 (flat) and Fla. 8442 (globe), as well as three near isogenic backgrounds. Recurrent parents, NILs, and F_1_ progeny resulting from their cross were then genotyped with markers specific to the *globe* locus.

To determine the extent to which the *globe* locus explains fruit shape across a broader collection of cultivated germplasm, a survey was conducted including large-fruited inbred lines from the UF/IFAS and NCSU breeding programs, and inbred lines and hybrids from multiple private sector breeding programs.

Further details about the development of the populations used for mapping and germplasm surveyed are provided in supplementary methods.

### DNA extraction

DNA was extracted using either a modified Cetyl Trimethylammonium Bromide (CTAB) method^42^ or a NaOH rapid DNA extraction method^43^. Samples were stored at 4 °C until PCR was performed. For either extraction method, tissue was collected from young leaves using a 3 mm diameter hole punch (2 punches for CTAB extraction, 1 punch for NaOH extraction).

### Whole-genome re-sequencing data, SNP detection, and gene annotations

Whole-genome resequencing data previously described^44^ was used to identify polymorphic loci for confirmation and fine mapping of the *globe* locus. In addition to SNPs, insertions/deletions (indels) were predicted from aligned read data using FreeBayes (version 0.9.16-1-gf46d24f) ^45^. Data were visualized using JBrowse 1.11.5 (ref. ^46^). Loci that were polymorphic between Fla. 8000 and Fla. 8111B were considered for marker development (described below). Haplotypes at additional polymorphic loci within the fine mapped interval were compared among other breeding lines for further sequence characterization. Sequences were compared for six flat-fruited breeding lines, Fla. 8000, Fla. 8624, Fla. 8820, Fla. 8916, Fla. 8924, and Fla. 8932; and for four globe-fruited breeding lines, Fla. 8111B, Fla. 8297, Fla. 8872B, and Fla. 8925. Gene annotations were based on version 4.1 of the International Tomato Annotation Group (ITAG4.1). Genome physical locations were based on the version SL4.0 of the Tomato Genome Assembly and the sequences were accessed through the Sol Genomics Network database (https://solgenomics.net/)^47^. The wild-type and mutant sequences of the candidate gene were translated into peptide sequences using the ExPASy Translate Tool (https://web.expasy.org/translate/).

### Marker development and testing

Selective genotyping for initial mapping was performed using the Illumina Infinium SolCAP tomato SNP array (Illumina Inc., San Diego, CA), which encompasses 7720 Single Nucleotide Polymorphism (SNP) sites distributed across the genome^48^. DNA samples were extracted using the CTAB method described above, and SNP chip processing was performed by the Michigan State University Potato Breeding and Genetics SNP genotyping facility. An Illumina iScan system was used for SNP chip processing, and SNP analysis was done using GenomeStudio 2.0 software (Illumina Inc., San Diego, CA).

Two hundred and thirty-eight F_2_ plants from the Fla. 8000 × Fla. 8111B cross were used for confirmation of the mapped flat/globe locus. Kompetitive allele-specific PCR (KASP; LGC Genomics, Beverly, MA), Cleaved Amplified Polymorphic Sequences (CAPS), derived Cleaved Amplified Polymorphic Sequences (dCAPS) markers, and an indel-based marker, were designed from polymorphisms spanning the mapped *globe* locus and used to genotype this population. Subsequent mapping and fine mapping relied upon High resolution Melting Analysis (HRM) and additional dCAPS markers. Marker protocols are described further in supplementary methods.

### Genetic data analysis

Monogenic inheritance of fruit shape was tested in the Fla. 8000 × Fla. 8111B F_6_ RIL population using a chi-square test for goodness of fit to 1:1 ratio of flat:globe phenotypes. Genotype data generated by the SolCAP SNP chip was used for initial mapping of the flat/globe fruit shape locus. Data were filtered, and SNPs identified as polymorphic between Fla. 8000 and Fla. 8111B and segregating among the 23 RILs were utilized for genetic map construction with JoinMap^®^ 4.1 using the Kosambi mapping function^49,50^. RIL phenotypes, along with SNP genotypes and calculated genetic positions were then used to perform inclusive composite interval mapping analysis with QTL IciMapping 4.1 (refs. ^51,52^). The ICIM-ADD mapping method was used with a scanning step of 1 cM and a probability in stepwise regression of 0.001 as implemented in the software.

To confirm monogenic inheritance of fruit shape in the Fla. 8000 × Fla. 8111B F_2_ population, the frequency of flat:globe phenotypes was tested for goodness of fit to a 3:1 ratio using a chi-square. Phenotypes of these F_2_ individuals along with their genotypes at eight markers were used to construct a genetic map and perform inclusive composite interval mapping as described above.

### Generation of CRISPR/Cas9 knock outs and measurements

#### Plant materials and growth conditions

Fla. 8059 seeds were sown directly in SunGro Germination mix soil in 72 count 6 pack trays and grown in a growth chamber under 16-h light/8-h dark photoperiod for 4 weeks under lithonia ibz 632 wd with ProLume eco-shield F32T8-850-ECO bulbs (32 watt/ 3,050 lumens full spectrum daylight/5000 K). Four weeks-old seedlings were transplanted into 6.06 L pots and were cultivated in an air-conditioned greenhouse under 16-h light/8-h dark photoperiod with daytime temperatures 75–80F (8am–6pm) and nighttime temperatures 60/65F (9pm–6am) in Athens, GA, USA.

#### Guide RNA design, CRISPR/Cas9 construct, and plant transformation

The gRNAs targeting Solyc12g006860 were designed using the CRISPR-P tool (http://cbi.hzau.edu.cn/cgi-bin/CRISPR)^53^. The gRNAs were selected to have no fewer than four mismatches within the coding region of potential off target genes. The CRISPR/Cas9 constructs were assembled using the NEBuilder Assembly of CRISPR vectors using ssDNA oligos as previously described^54^. Electroporation was used to introduce the final binary vectors into *Agrobacterium tumefaciens* strain LBA4404, which was kindly provided by Dr. Joyce Van Eck, Cornell University. The LBA4404 harboring the binary vector was used for the transformations of Fla. 8059. The genetic transformations were performed as described^55^. The gRNAs were cloned and transgenic plants were genotyped using primers listed in Table S2. A single T0 plant (with two gRNA targeting the globe gene, both heterozygous) was selfed. Five T1 homozygous mutant plants were selected and used for measurements along with five WT Fla. 8059.

#### Fruit shape and pedicel length measurements

Ripe fruits were halved and scanned at 200dpi using an HP Scanjet G4050. These images were analyzed using Tomato Analyzer to determine shape index (Length/width)^56^. Pedicel length was measured using calipers. At least three (and up to eleven) measurements were performed for each of five WT plants and five mutant plants. The average of the shape index and pedicel length per plant was determined and used to perform a t-test to look into statistically significant differences.

### *In silico* survey of germplasm

The distribution of the globe allele across a broad collection of wild and domesticated tomato germplasm was determined *in silico* using BLAST search^57^ against publicly available tomato sequencing projects^58–60^. Data was retrieved from the Sequencing Read Archive (https://www.ncbi.nlm.nih.gov/sra). A 61-nucleotide sequence containing the single base pair insertion in the globe allele (consisting of 30 bp upstream and downstream of the insertion) was used as a query. Blastn program was chosen, leaving all other parameters to default values.

### Plant materials for phenotypic characterization

The three flat NILs described previously along with the globe recurrent parents and corresponding backcross F_1_s were used for the characterization trials. The trials were conducted during three seasons (fall 2017, spring 2018, and fall 2018) at the UF/IFAS Gulf Coast Research and Education Center in Balm, FL. The plants were established in the field in 12-plant plots arranged in a randomized complete block design with four replicates. Eight plants were harvested from each plot. Due to pollination problems, the backcross F_1_ for the Fla. 8022 background was not evaluated in spring 2018.

### Fruit size, shape and marketability measurements

Fruit weight was assessed from four to five weekly harvests of vine-ripened fruit in each of the three trials. The fruit from each experimental unit were graded for marketability and size according to the US Standards for Grades of Fresh Tomatoes (USDA-Agricultural Marketing Service). The fruit weight was obtained by averaging the total weight of marketable fruit over the total number of fruits per plot. Culled fruit included, but were not limited to, fruit with radial cracking, weather check, zippering, zippering with windows, and rough scars. Radial cracking and weather check are illustrated in Fig. S1. Culled fruits were grouped according to the defect, and fruits in each group were counted and weighed. Marketable fruits were sized into small (S), medium (M), large (L), and extra-large (XL) categories, and fruits of each category were counted and weighed. The average weight per plant for each marketable size category was used for statistical analysis. For the cull categories, the percentage of total harvest was used for analysis.

For fruit shape, 24 mature fruit per plot, from the second to fourth harvest, were randomly selected and cut in half longitudinally. One half of each fruit was scanned into a 200-dpi (dots per square inch) image. Several fruit shape attributes were measured from images with the Tomato Analyzer 2.2 software^56^. The default settings of the software were used, and boundaries and end points were corrected as needed. The attributes assessed in this study are listed in Table S3. The average value of each attribute per plot was used for statistical analysis.

Further details on data collection for plant biomass and fruit pedicel are described in the supplementary methods.

### Statistical analysis

A linear mixed effect analysis was performed to assess the association of the genotype at the *globe* locus with each of the traits measured. The model was defined as:

$$y_{pqij} = \mu _{ij} + L_p + b_{pq}(L) + \varepsilon _{pqij}$$

where $L_p$, $b_{pq}(L)$, and *ε*_*pqij*_ represent the variance due to season, block (nested within season), and the residual, respectively, and conform the design structure effects, hence were set as random. On the other hand, *μ*_*ij*_ is the mean structure for the fixed effects given by:

$$\mu _{ij} = \mu + \alpha _i + \beta _j + \alpha \beta _{ij}$$

where *μ* is the overall mean, *α*_*i*_ is the main effect for the *ith* genotype at the fruit shape locus (globe line G/G, flat NIL F/F, F_1_ F/G), *β*_*j*_ is the main effect for the *jth* genetic background (Fla. 7776, Fla. 8022, Fla. 8735), and *αβ*_*ij*_ is the interaction between genotype and background. The design structure effects have nothing to do with the mean structure and interactions between season and treatments were not included for this reason^61^.

Since it is known that, in tomato, fruit yield and marketability usually show a high variation across seasons, and fruit disorders like weather check are known to be highly dependent on weather, the analysis of the marketability traits was made individually for each season, hence the *L*_*p*_, and *b*_*pq*_(*L*) terms in the design structure of the model were dropped and *b*_*q*_, representing the block effect, was added instead.

The R programing language (R Core Team 2017) was used to perform the analysis^62^. The *lmer* function of the lme4 package^63^ was used to fit the model. The model assumptions were checked by visually inspecting the QQ plot, histogram, and distribution of the standardized residuals. Weather check data in both fall seasons was log-transformed in order to meet the model assumptions.

The model results were later entered into the *lsmeans* function to calculate the fixed-effects estimates and *p*-values of the pairwise differences adjusting with the Tukey HSD method (*P* ≤ 0.05). The significance letters were generated with the *cld* function. Both functions belong to the lsmeans package^64^. The pairwise differences were calculated within and among backgrounds.

## Supplementary information

Supplementary file 1 - marked up

Supplementary file 1

Supplementary file 2

## Data Availability

Supporting data for this research may be requested from the corresponding author.
