# Supplementary material for: Identification and characterization of GLOBE, a major gene controlling fruit shape and impacting fruit size and marketability in tomato
Source: Hortic Res. 2021 Jun 1;8:138. doi: 10.1038/s41438-021-00574-3 (PMC8169893; doi:10.1038/s41438-021-00574-3)
Supplement: Supplementary file 1 — marked up [file 41438_2021_574_MOESM1_ESM.docx]

## Supplementary methods

### Plant Materials for mapping and fine mapping

A Recombinant Inbred Line (RIL) population from the cross between Fla. 8000 (flat shaped line) and Fla. 8111B (globe shaped line) was developed by advancing 83 F_2_ plants by single seed decent to the F_6_ generation. Fla. 8000 and Fla. 8111B are large-fruited, fresh-market breeding lines from the UF/IFAS tomato breeding program. Six plants of each RIL were planted to the field and scored at maturity as having flat or globe fruit shape.

Fine mapping of the locus was conducted in populations from the same cross following a map-based approach. Recombinant plants resulting from crossing-over within the target interval were identified in F_3_ and F_4_ populations segregating for the locus, and each recombinant plant was advanced by selfing. Progeny of recombinants were screened with appropriate markers to identify individuals which were homozygous for the recombined segments in order to develop a population of RILs for fine mapping (FM-RILs). Four plants per FM-RIL were planted to the field for subsequent phenotyping of fruit shape, and FM-RILs were also genotyped with markers saturating the mapped interval.

Flat near isogenic lines (NILs) for each of the globe-fruited parents, Fla. 8735, Fla. 7776, and Fla. 8022, were developed by introgression of the flat fruit shape trait from the donor parents, Fla. 8059, Fla. 7946, and Fla. 8021, respectively. All are fresh-market large-fruited breeding lines from the UF/IFAS breeding program. Using a modified backcrossing approach, flat fruit shape was phenotypically selected after each cross and backcross in the F_2_ and/or F_3_ generation, without the use of markers. The trait was thereby advanced to the BC_3_ generation for each NIL. Thus, in theory, each NIL was 93.75% genomically identical to its recurrent parent. F_1_s were created between each globe breeding line and its respective flat NIL.

The modern tomato germplasm survey included 176 large-fruited inbred lines with flat (129 lines) or globe (47 lines) fruit shape, 28 cherry, grape and plum lines, and 35 large-fruited hybrids with flat fruit shape (Tables S4 and S5). Fruit shape phenotypes of materials from UF/IFAS and NCSU were collected from germplasm releases or breeding program records. Phenotypes of private sector lines were reported by industry breeders and verified in the field at fruit maturity. All material included in the germplasm survey was genotyped with one or more markers corresponding to the flat/globe locus.

### Marker Development and Testing

Kompetitive allele specific PCR (KASP; LGC Genomics, Beverly, MA), Cleaved Amplified Polymorphic Sequences (CAPS), derived Cleaved Amplified Polymorphic Sequences (dCAPS) markers, and an indel-based marker, were designed from polymorphisms spanning the mapped flat/globe locus and used for genotyping this population. Both CAPS and dCAPS markers were designed using dCAPS Finder 2.0 (<http://biology4.wustl.edu/dcaps/>) in combination with the NEBCutter tool (New England Biolabs, Inc., Ipswich, MA). Specific primer pairs were designed using Primer3 software (<http://frodo.wi.mit.edu/>). PCR was performed in a final volume of 20 μL containing 40 ng template genomic DNA, 1 × PCR buffer, 2 mM MgCl2, 0.1 mM dNTPs, and 0.1 μM for each primer. The following PCR conditions were used: an initial denaturing step at 94 °C (2 min), then 35 cycles of 94 °C (30 s), 52–56 °C (30 s) and 72 °C (30 s), followed by a final elongation step at 72 °C (5 min). PCR products were visualized on 3% TBE agarose gel by ethidium bromide staining. The KASP assays were performed in a final reaction volume of 5 μL containing of 1× low ROX KASP master mix V4.0 (LGC Genomics, LLC, Beverly, MA, USA), 0.106 μL of the KASP assay primer mix (allele specific primers at 12 μM and the common reverse primer at 30 μM) and 20-50 ng genomic DNA. The following PCR conditions were used: an initial Taq activation step at 94 ºC for 15 min, followed by 10 cycles of touch down PCR from 65 ºC to 57 ºC with 0.8 ºC decrease per cycle, then followed by 40 cycles of 94 ºC (20 s) and 57 ºC (1 min). PCR endpoint detection was performed using the Infinite® M200 Pro microplate reader (Tecan Group Ltd, Männedorf, Switzerland). Visualization of the clusters with the SNP allele callings was obtained by KlusterCaller software v.3.4.1.36 (LGC Genomics, Hoddesdon, Herts, UK).

The primers for HRM markers were designed using the IDT PrimerQuest tool (<https://www.idtdna.com/PrimerQuest/Home/Index>). The PCR protocol for these markers was as follows: Five µL PCR reactions were comprised of 0.5 µL of DNA solution (1-10 ng/ µL), 2 µL of AccuStart® II PCR SuperMix 2X (Quatabio, Beverly, MA), 0.25 µL of EvaGreen® Dye 2X (Biotium, Hayward, CA), 0.05 µL of each forward and reverse primers (0.1 mM), and 2.15 µL of HPLC water. For probe-based markers the PCR protocol was modified as follows: the amount of reverse primer was doubled (0.1 µL); the amount of forward primer was diluted to one-fifth (0.01 µL); 0.1 µL of a 3’-phosphate-blocked probe (0.1 mM) was added to the PCR solution; and the amount of water was adjusted to maintain 5 µL total volume. PCR amplification was performed using a Mastercycler® Pro 384 (Eppendorf, Hauppauge, NY) with the following temperatures and durations: 94 °C for 30 seconds for initial denaturation; followed by 40 cycles of denaturation at 94 °C for 5 seconds, annealing for 10 seconds, and extension at 72 °C for 15 seconds; then a final extension at 72 °C for 1 minute. Annealing temperatures are specific for each marker and presented in Table S1. Digested products of dCAPS markers were detected on 3% agarose gels stained with ethidium bromide. HRM markers were detected by melting curve analysis using a LightCycler® 480 Instrument II (Roche, Pleasanton, CA).

### Effects on Plant Biomass and Fruit Pedicel

Plant biomass was measured at final harvest in fall 2017 and at first harvest in spring and fall 2018. Each season, eight plants per plot were cut at the soil level, remaining fruits were removed, and fresh weight was recorded. The average weight of the eight plants of each experimental unit was used for the statistical analysis.

With respect to fruit pedicel, three pedicels from each of five plants were sampled from each experimental unit. The length from the base of the pedicel (sepals’ level) to the joint of the pedicel, and the width at half the length, were measured with a caliper. Only pedicels from mature green to ripe fruits were used for sampling. The average of the 15-pedicel measurements on each experimental unit was used for statistical analysis.

## Supplementary tables

Table S1. Molecular markers located on chromosome 12 and used for fine mapping the flat/globe fruit shape locus.

| Marker | Position^1^ | Polymorph. detected | Assay^2^ | RE^3^ | Primers (5' to 3') |  | |  |
| --- | --- | --- | --- | --- | --- | --- | --- | --- |
|  |  |  |  |  | Forward | Reverse | Annealing Temp. | |
| 16EP267 | 390,069 | SNP | KASP | NA | reference allele_c_hex: gaaggtcggagtcaacggattgtacgaaacacaaaattccgcc alternative allele_t_fam: gaaggtgaccaagttcatgctatgtacgaaacacaaaattccgct | ggataataatggcagttggaatgg |  | |
| 15EP210 | 723,874 | SNP | dCAPS | BsaHI | aacacagcttgggcttcaac | cagcttgtgatagtgtgaaggtgac |  | |
| 15EP214 | 936,163 | 8-bp indel | Indel | NA | tttggtagctgattagagtcatgt | tttgatgtgtgtttgcaatttaga |  | |
| M1 | 936,308 | SNP | HRM | NA | aaataacaccgcacacatag | ttatggaatcatgtgatatcgg | 56°C | |
| M1.1 | 971,520 | SNP | HRM | NA | acaagccaagagcatacaa | tgttgggattatggatcacc | 55°C | |
| M1.2 | 986,979 | SNP | HRM | NA | ctaagtaagaacatccctttcattc | cctgaacttgggacattaaga | 57°C | |
| M2 | 1,021,040 | SNP | HRM | NA | tcataatcatcgtctactgtattt | acatcacaacctcaacttatc | 50°C | |
| 16EP95 | 1,021,750 | SNP | CAPS | HaeIII | caaattatcgatttcaatttttca | tgaccttaggtattaatgtgttagcc |  | |
| M2.1 | 1,031,490 | SNP | dCAPS | NsiI | gatgttgaatgtaaatatgttttttta | ggccaatgtaacacctaat | 50°C | |
| M3 | 1,306,260 | 1-bp indel | HRM^4^ | NA | acgattatcgccacaatataaacac | gagtcaaccgttgtttgaagatg | 60°C | |
| 16EP133 | 1,423,513 | SNP | dCAPS | HinfI | atggcctagttcctactttgctatt | actgttttcaaaagatacttaaatcaagtgatt |  | |
| M4 | 1,427,944 | SNP | HRM | NA | aaaccgattaagtagcaatgattag | tctgaacctgataaaggaatatgt | 52°C | |
| M4.1 | 1,894,619 | SNP | HRM | NA | acagttaaattgaaacaaatga | tcaatagaaagtaattaaatcgaa | 47°C | |
| 16EP219 | 2,049,214 | SNP | KASP | NA | reference allele_a_hex: gaaggtcggagtcaacggattttgtcccctctcctggttca alternative allele_g_fam: gaaggtgaccaagttcatgcttgtcccctctcctggttcg | agtcgaccccagtgtttccag |  | |

Table S1. Continued

| Marker | Position^1^ | Polymorph. detected | Assay^2^ | RE^3^ | Primers (5' to 3') |  | |
| --- | --- | --- | --- | --- | --- | --- | --- |
|  |  |  |  |  | Forward | Reverse | Annealing Temp. |
| M5 | 2,049,214 | SNP | HRM | NA | tgcttcaatgatcacaccat | ctaacacaggcgccattag | 57°C |
| 16EP276 | 2,587,614 | SNP | KASP | NA | reference allele_c_hex: gaaggtcggagtcaacggattgtcataatcttatccatcgatccgac alternative allele_t_fam: gaaggtgaccaagttcatgctgtcataatcttatccatcgatccgat | taatcgtgatttgcttttgaacctc |  |
| 16EP322 | 2,877,860 | SNP | KASP | NA | reference allele_c_hex: gaaggtcggagtcaacggattaaaatggatcggtttggcc alternative allele_t_fam: gaaggtgaccaagttcatgctcaaaatggatcggtttggct | ttggtgatctagagttccaatgattt |  |

^1^Chromosome 12 physical position (in bp) based on the SL4.0 tomato genome assembly; ^2^KASP: Kompetitive allele specific PCR, dCAPS: derived cleaved amplified polymorphic sequences, CAPS: Cleaved amplified polymorphic sequences, Indel: Insertion or deletion, HRM: High Resolution Melting Analysis; ^3^RE: Restriction enzyme; ^4^Marker M3 is a probe-based melting analysis, probe sequence (5’ to 3’): tgctgatgctacttcatcctcaatatgg (3’ phosphate modified)

Table S2: Primer Sequences for the gRNAs used in CRISPR editing (18EP960 and 18EP961). Primer sequences for cloning using NEBuilder Assembly (17EP783,29,30,32,37,39). Primer sequences used to genotype the two gRNAs (20EP762/763 and 19EP371/372) and for presence of Cas9 (14EP426/427) in transgenic plants.

| Primer Name | Sequence | Notes |
| --- | --- | --- |
| 18EP960 | TCAAGCGAACCAGTAGGCTT GTTGCTAAACAAGGTATTAG GTTTTAGAGCTAGAAATAGC | gRNA 1 with overlap for cloning |
| 18EP961 | TCAAGCGAACCAGTAGGCTT GAAGTAGCATCAGCACGGTT GTTTTAGAGCTAGAAATAGC | gRNA 2 with overlap for cloning |
| 17EP783 | GTGCTTTGGATCGATCTGCCCAATGCCTATCTTATATGATCAATGAGG | SpeI_MtU6F |
| 17EP30 | GAGAATGGATGCGAGTAATGAAAAAAAGCACCGACTCGGTG | SpeI_Scaffold R |
| 17EP29 | CATTACTCGCATCCATTCTCATGCCTATCTTATATGATCAATGAGG | UNS1_MtU6 F |
| 17EP32 | TCCGTCTACGAACTCCCAGCAAAAAAAGCACCGACTCGGTG | UNS2_ScaffoldR |
| 17EP37 | ACATGCACCTAATTTCACTAGATGT | Sequencing p201 F |
| 17EP39 | CGCGCCGAATTCTAGTGATCG | Sequencing p201 R |
| 20EP762 | TTGGCCTTGCCAAACAAAC | Sequence gRNA 1 |
| 20EP763 | GCATAGTTTGAGAAGAAGCCTTTAG | Sequence gRNA 1 |
| 19EP371 | TGGGCTTGGAGTACGAC | Sequence gRNA 2 |
| 19EP372 | GGCATGATGATGACTCTACTTG | Sequence gRNA 2 |
| 14EP426 | \| CCGACGCTAACCTCGATAAG \| \| --- \| | Cas9 |
| 14EP427 | \| CGAGCTGAGAGAGGTCGATT \| \| --- \| | Cas9 |

Table S3. Fruit shape traits measured in this study using Tomato Analyzer.

| Group | Attribute | Unit | Description | |
| --- | --- | --- | --- | --- |
| Basic Measurements | Height | mm | Height at mid width | |
|  | Width | mm | Width at mid height | |
|  | Area | mm^2^ | Area | |
|  | Perimeter | mm | Perimeter | |
| Fruit shape index | Fruit shape index | ratio | Height/Width | |
| Proximal fruit end shape | Shoulder height | ratio | Height of shoulder/total height | |
|  | Proximal angle at 10% of perimeter | degrees | Angle | |
|  | Proximal angle at 20% of perimeter | degrees | Angle | |
|  | Proximal indentation area | ratio | Indentation area/total area | |
| Distal fruit end shape | Distal angle at 10% of perimeter | degrees | Angle | |
|  | Distal angle at 20% of perimeter | degrees | Angle | |
|  | Distal indentation area | ratio | Indentation area/total area | |
| Blockiness descriptors | Proximal fruit blockiness | ratio | Proximal width/Width |  |
|  | Distal fruit blockiness | ratio | Distal width/width |  |
|  | Fruit shape triangle | ratio | Proximal width/Distal width | |
| Asymmetry | Ovate | index | How top heavy is the fruit | |
|  | Position of widest width | index | Position of widest diameter along the Vertical axis, 0 proximal, 1 distal end | |
|  | Vertical asymmetry | index | 0 is a perfect symmetric shape | |
|  | Horizontal asymmetry | index | 0 is a perfect symmetric shape | |
| Homogeneity | Circular | R^2^ | Fitting precision R^2^ | |
|  | Elliptic | R^2^ | Fitting precision R^2^ | |
|  | Rectangular | ratio | Area of inscribing/enclosing rectangles | |

Table S4. Fruit shape phenotype of tomato inbred lines from multiple public and private breeding programs and haplotypes spanning the flat/globe fruit shape locus.

| Phenotype | Line | Source | Haplotype No. | No. of Lines | Fruit shape locus genotypes^1^ | | | | | | | | |
| --- | --- | --- | --- | --- | --- | --- | --- | --- | --- | --- | --- | --- | --- |
|  |  |  |  |  | M1.1 | M1.2 | | M2 | | M3 | | M4 | M4.1 |
| Flat | EZ1, EZ7 | Enza Zaden | 1 | 71 | G/G | | G/G | | G/G | | F/F | G/G | F/F |
|  | EDR223, EDR228 | HM.CLAUSE |  |  |  |  |  |  |  |  |  |  |  |
|  | 1724, 1757, 4468, 4528, 4529, 4532, 4600 | Lipman Produce |  |  |  |  |  |  |  |  |  |  |  |
|  | NC 161L, NC10122(x)-04-F6 | NCSU |  |  |  |  |  |  |  |  |  |  |  |
|  | 1677791, 1677792, 1677794, 1677795, 1677796, 1677797, 1677798, 1677808, 1677809, 1677810, 1677811, 1677812, 1677813, 1677814, 1677815, 1677834, 1677835, 1677836, 1677837, 1677838, 1677839, 1677840, 1677841, 1677845, 1677846, 1677848, 1701687, 1701762, 1701764, 1701768, 1701772, 1701775, 1701776, 1701780, 1701781, 1701782, 1701783, 1701784, 1701785, 1701786, 1701787, 1701792, 1701793 | Sakata |  |  |  |  |  |  |  |  |  |  |  |
|  | Fla. 7481, Fla. 7547, Fla. 7771, Fla. 7775, Fla. 7781, Fla. 7804, Fla. 7946, Fla. 8044, Fla. 8059, Fla. 8249, Fla. 8539, Fla. 8543, Fla. 8653, Fla. 8820, Fla. 8822 | UF/IFAS |  |  |  |  |  |  |  |  |  |  |  |
|  | EZ2 | Enza Zaden | 2 | 33 | F/F | | F/F | | F/F | | F/F | G/G | F/F |
|  | EDR002, EDR220, EDR227, EDR229 | HM.CLAUSE |  |  |  |  |  |  |  |  |  |  |  |
|  | 1717, 1718, 1725, 1726, 1729, 1730, 1743, 1749, 4530, 4531, 4533, 4534 | Lipman Produce |  |  |  |  |  |  |  |  |  |  |  |
|  | 1677799, 1677800, 1677842, 1677843, 1701685, 1701686, 1701794, 1701788, 1701795 | Sakata |  |  |  |  |  |  |  |  |  |  |  |
|  | Fla. 7171, Fla. 7236, Fla. 7600, Fla. 7770, Fla. 8233, Fla. 8476, MH1 | UF/IFAS |  |  |  |  |  |  |  |  |  |  |  |
|  | EZ3, EZ4, EZ8, EZ9, EZ12 | Enza Zaden | 3 | 8 | F/F | | F/F | | F/F | | F/F | G/G | G/G |
|  | 1735 | Lipman Produce |  |  |  |  |  |  |  |  |  |  |  |
|  | 1701771, 1701763 | Sakata |  |  |  |  |  |  |  |  |  |  |  |

Table S4. Continued

| Phenotype | Line | Source | Haplotype No. | No. of Lines | Fruit shape locus genotypes^1^ | | | | | | | | |
| --- | --- | --- | --- | --- | --- | --- | --- | --- | --- | --- | --- | --- | --- |
|  |  |  |  |  | M1.1 | M1.2 | | M2 | | M3 | | M4 | M4.1 |
| Flat | EZ5, EZ6, EZ10, EZ11 | Enza Zaden | 4 | 8 | G/G | | G/G | | G/G | | F/F | G/G | G/G |
|  | 4496 | Lipman Produce |  |  |  |  |  |  |  |  |  |  |  |
|  | 1677847 | Sakata |  |  |  |  |  |  |  |  |  |  |  |
|  | Fla. 8021, Fla. 8814 | UF/IFAS |  |  |  |  |  |  |  |  |  |  |  |
|  | Fla. 8000, 8561B | UF/IFAS | 5 | 2 | F/F | | F/F | | F/F | | F/F | F/F | F/F |
|  | 1677793 | Sakata | 6 | 2 | F/F | | G/G | | G/G | | F/F | G/G | F/F |
|  | Fla. 8624 | UF/IFAS |  |  |  |  |  |  |  |  |  |  |  |
|  | Fla. 8570 | UF/IFAS | 7 | 1 | G/G | | G/G | | F/F | | F/F | G/G | F/F |
|  | NC 1SC | NCSU | 8 | 2 | G/G | | F/F | | G/G | | F/F | G/G | F/F |
|  | 1728 | Lipman Produce |  |  |  |  |  |  |  |  |  |  |  |
|  | 1701788 | Sakata | 9 | 1 | F/F | | F/F | | F/F | | F/F | G/G | F/F |
|  | Fla. 8608 | UF/IFAS | het 1,2 | 1 | F/G | | F/G | | F/G | | F/F | G/G | F/F |
| Globe | EZ11 (2) | Enza Zaden | 10 | 40 | G/G | | G/G | | G/G | | G/G | G/G | G/G |
|  | 1719, 1721, 1742, 1745, 1746, 1750, 1751, 1753, 1754 | Lipman Produce |  |  |  |  |  |  |  |  |  |  |  |
|  | NC 84173 | NCSU |  |  |  |  |  |  |  |  |  |  |  |
|  | 1677807, 1701765, 1701766, 1701767, 1701769, 1701773, 1701778, 1701779 | Sakata |  |  |  |  |  |  |  |  |  |  |  |
|  | Fla. 7060, Fla. 7776, Fla. 7907B, Fla. 8022, Fla. 8109, Fla. 8111B, Fla. 8124C, Fla. 8293, Fla. 8297, Fla. 8344, Fla. 8352, Fla. 8599, Fla. 8626, Fla. 8629B, Fla. 8735, Fla. 8869, Fla. 8872B, Fla. 8925, Fla. 8923D, Fla. 8979, Fla. 8980, Fla. 8981 | UF/IFAS |  |  |  |  |  |  |  |  |  |  |  |
|  | 1722, 4395 | Lipman Produce | 11 | 2 | G/G | | G/G | | G/G | | G/G | G/G | F/F |

Table S4. Continued

| Phenotype | | Line | Source | | Haplotype No. | | | No. of Lines | Fruit shape locus genotypes^1^ | | | | | | | | | | | | | | |
| --- | --- | --- | --- | --- | --- | --- | --- | --- | --- | --- | --- | --- | --- | --- | --- | --- | --- | --- | --- | --- | --- | --- | --- |
|  |  |  |  |  |  |  |  |  | M1.1 | | | M1.2 | | | | M2 | | M3 | | | M4 | M4.1 | |
| Globe | | NC 714 | NCSU | | 12 | | | 1 | G/G | | | | | | G/G | | G/G | | | G/G | F/F | G/G | |
|  |  | 1723, 1727, 1732 | Lipman Produce | | 13 | | | 3 | F/G | | | | | | F/F | | F/G | | | G/G | G/G | F/G | |
|  |  | 1748 | Lipman Produce | | 14 | | | 1 | G/G | | | | | | F/G | | G/G | | | G/G | G/G | G/G | |
| Cherry | | Fla. 701 | UF/IFAS | | 2 | | | 1 | F/F | | | | | | F/F | | F/F | | | F/F | G/G | F/F | |
|  |  | Fla. 8737 | UF/IFAS | | 6 | | | 1 | F/F | | | | | | G/G | | G/G | | | F/F | G/G | F/F | |
| Grape | | NC5Grape, NC6Grape | NCSU | | 1 | | | 2 | G/G | | | | | | G/G | | G/G | | | F/F | G/G | F/F | |
|  |  | NC22L-1(2008), NC4Grape | NCSU | | 2 | | | 2 | F/F | | | | | | F/F | | F/F | | | F/F | G/G | F/F | |
| Plum | 47L-2W(2010), NC 14180_2B_F4_2, NC 25P | | | NCSU | 1 | | 5 | | | G/G | | | G/G | | | | G/G | | F/F | | G/G | F/F |  |
|  | 1701689, 1701690 | | | Sakata |  |  |  |  |  |  |  |  |  |  |  |  |  |  |  |  |  |  |  |
|  | STC8324 | | | HM.CLAUSE | | het 1,10 | | 1 | | | G/G | | | G/G | | | G/G | | F/G | | G/G | F/F |  |
|  | 4582 | | | Lipman Produce | | 2 | | 3 | | | F/F | | | F/F | | | F/F | | F/F | | G/G | F/F |  |
|  | 1701691, 1701692 | | | Sakata | |  |  |  |  |  |  |  |  |  |  |  |  |  |  |  |  |  |  |
|  | 4318 | | | Lipman Produce | | 10 | | 5 | | | G/G | | | G/G | | | G/G | | G/G | | G/G | G/G |  |
|  | 1677869, 1701790 | | | Sakata | |  |  |  |  |  |  |  |  |  |  |  |  |  |  |  |  |  |  |
|  | Fla. 8344, Fla. 8517 | | | UF/IFAS | |  |  |  |  |  |  |  |  |  |  |  |  |  |  |  |  |  |  |
|  | NC 30P, NC946 | | | NCSU | | 11 | | 4 | | | G/G | | | G/G | | | G/G | | G/G | | G/G | F/F |  |
|  | 1677871 | | | Sakata | |  |  |  |  |  |  |  |  |  |  |  |  |  |  |  |  |  |  |
|  | Fla. 726 | | | UF/IFAS | |  |  |  |  |  |  |  |  |  |  |  |  |  |  |  |  |  |  |
|  | Fla. 8083 | | | UF/IFAS | | 10 or 11 | | 1 | | | G/G | | | G/G | | | G/G | | G/G | | G/G | ? |  |
|  | STC8332 | | | HM.CLAUSE | | het 1,2 | | 1 | | | F/G | | | F/G | | | F/G | | F/F | | G/G | F/F |  |
|  | STC8320 | | | HM.CLAUSE | | het 2,4 | | 1 | | | F/G | | | F/G | | | F/G | | F/F | | G/G | F/G |  |
|  | STC8331 | | | HM.CLAUSE | | het 2,11 | | 1 | | | F/G | | | F/G | | | F/G | | F/G | | G/G | F/F |  |

^1^F/F, Homozygous for the flat allele; F/G, Heterozygous; G/G, Homozygous for the globe allele

Table S5. Genotypes of commercial hybrids genotyped with the M3 marker linked to the flat/globe locus.

| Hybrids | Genotype^1^ | Source |
| --- | --- | --- |
| Bejo 3064, Emmylou, Jolene | F/F | Bejo Seeds, Inc. |
| Bejo 3096, Resolute | F/G | Bejo Seeds, Inc. |
| BHN 1006, BHN 1048 | F/F | BHN Seed |
| Skyway | F/F | Enza Zaden |
| Red Bounty, Red Defender | F/G | HM.CLAUSE |
| XTM2263, XTM2273 | F/F | Sakata Seed America |
| Charger, Grand Marshall, XTM2255, XTM2261, XTM3309 | F/G | Sakata Seed America |
| Cypress, Dixie Red, Sanibel, Southern Ripe | F/F | Seminis Vegetable Seeds |
| Crown Jewel, FL 47, Phoenix, Solar Set, SV7101TD, SV7631TD | F/G | Seminis Vegetable Seeds |
| Brickyard, Ridgerunner, Sebring, Soraya | F/F | Syngenta |
| Seventy III | F/G | Syngenta |
| VTR2170025 | F/F | VoloAgri |
| VTR2170023, VTR2170024 | F/G | VoloAgri |

^1^F/F, Homozygous for the flat allele; F/G, Heterozygous; G/G, Homozygous for the globe allele

Table S6. Annotated genes in the 392 kb fine-mapped interval between markers M2.1 and 16EP133.

| Locus | Annotation^1^ |
| --- | --- |
| Solyc12g006520.2.1 | Terpene cyclase/mutase family member |
| Solyc12g006530.2.1 | Terpene cyclase/mutase family member |
| Solyc12g006540.2.1 | Signal peptidase I |
| Solyc12g006550.2.1 | Ribosome biogenesis regulatory protein homolog |
| Solyc12g006560.2.1 | Early nodulin-93 |
| Solyc12g006570.2.1 | Sesquiterpene synthase |
| Solyc12g006575.1.1 | Pentatricopeptide repeat-containing family protein |
| Solyc12g006590.3.1 | Zinc finger protein-like protein |
| Solyc12g006600.3.1 | Zinc finger protein-like protein |
| Solyc12g006610.3.1 | Zinc finger (Ran-binding) family protein |
| Solyc12g006620.2.1 | Zinc finger transcription factor 72 |
| Solyc12g162400.1.1 | Plant/F18B13-26 protein |
| Solyc12g006630.2.1 | Dynein light chain-like protein |
| Solyc12g006640.2.1 | Lactoylglutathione lyase/glyoxalase I family protein |
| Solyc12g006650.2.1 | RNA polymerase II transcription mediator |
| Solyc12g006660.2.1 | testis- and ovary-specific PAZ domain protein |
| Solyc12g006670.3.1 | Binding protein |
| Solyc12g006680.2.1 | Early nodulin 93 protein |
| Solyc12g006690.2.1 | Protein kinase PINOID 2 |
| Solyc12g006695.1.1 | Unknown protein |
| Solyc12g006700.3.1 | DUF1644 domain-cointaining protein |
| Solyc12g160480.1.1 | DUF1644 domain-cointaining protein |
| Solyc12g006710.3.1 | Tudor domain protein |
| Solyc12g006720.1.1 | Unknown protein |
| Solyc12g006730.1.1 | Unknown protein |
| Solyc12g006740.1.1 | Unknown protein |
| Solyc12g006750.1.1 | Unknown protein |
| Solyc12g006760.1.1 | Unknown protein |
| Solyc12g006770.1.1 | Unknown protein |
| Solyc12g006780.1.1 | Unknown protein |
| Solyc12g006790.3.1 | Argonaute10b |
| Solyc12g006800.2.1 | myb family trancsription factor EFM-like |
| Solyc12g006805.1.1 | SOUL heme-binding family protein |
| Solyc12g006820.1.1 | 3-ketoacyl-CoA synthase |
| Solyc12g006830.2.1 | Histone H2A |
| Solyc12g006840.2.1 | Lectin protein kinase family protein |
| Solyc12g006850.2.1 | LELKT1GEN L. esculentum potassium channel |
| Solyc12g006860.2.1 | brassinosteroid hydroxylase |
| Solyc12g006870.2.1 | Alpha/beta-Hydrolases superfamily protein |
| Solyc12g006880.2.1 | PPPDE thiol peptidase family protein |

Table S6. Continued

| Locus | Annotation^1^ |
| --- | --- |
| Solyc12g006890.2.1 | Unknown protein |
| Solyc12g006920.2.1 | Serine/threonine protein phosphatase 2A regulatory subunit B |
| Solyc12g006930.2.1 | Acyl-[acyl-carrier protein] hydrolase |
| Solyc12g006940.2.1 | RING/U-box superfamily protein |
| Solyc12g006950.2.1 | Sulfate transporter-like protein |
| Solycg12006960.2.1 | Sulfate transporter 3.2 |
| Solyc12g006970.1.1 | Unknown protein |
| Solyc12g006973.1.1 | Leucine-rich repeat family protein |
| Solyc12g006977.1.1 | Unknown protein |
| Solyc12g006980.2.1 | Leucine-rich repeat receptor-like protein kinase family |

^1^Based on the annotation version ITAG4.1

Table S7. Marketability results.

|  | Fall 2017 | | | | | | | | | | Spring 2018 | | | | | | | | | | | | | | | | | | | Fall 2018 | | | | | | | | | | | | | | | |
| --- | --- | --- | --- | --- | --- | --- | --- | --- | --- | --- | --- | --- | --- | --- | --- | --- | --- | --- | --- | --- | --- | --- | --- | --- | --- | --- | --- | --- | --- | --- | --- | --- | --- | --- | --- | --- | --- | --- | --- | --- | --- | --- | --- | --- | --- |
|  | Fla. 7776 | | Fla. 8022 | | | Fla. 8735 | | | | SE | Fla. 7776 | | | | Fla. 8022 | | | | | Fla. 8735 | | | | | SE | | | | | Fla. 7776 | | | Fla. 8022 | | | | | Fla. 8735 | | | | | SE | | |
| Marketable (Kg/plant) | | | | |  | |  | |  | |  |  |  | | |  | |  | | |  | |  | | |  | |  | | |  | | |  | |  | | |  | |  | | |  | |
| F/F | 0.189 | a | 2.222 | ab | | 1.540 | | a | | 0.169 | 5.014 | | | ab | | | 5.950 | | a | | | 6.385 | | b | | | 0.326 | | 0.873 | | | a | | | 4.943 | | a | | | 3.560 | | a | | | 0.329 |
| F/G | 0.743 | ab | 2.677 | b | | 1.664 | | a | | 0.169 | 5.643 | | | b | | |  | |  | | | 6.860 | | b | | | 0.326 | | 1.818 | | | ab | | | 5.261 | | a | | | 3.994 | | a | | | 0.329 |
| G/G | 1.168 | b | 2.002 | a | | 1.934 | | a | | 0.169 | 4.207 | | | a | | | 5.039 | | a | | | 4.358 | | a | | | 0.326 | | 2.588 | | | b | | | 4.439 | | a | | | 4.222 | | a | | | 0.329 |
| Extra-large (Kg/plant) | | | | |  | |  | |  | |  |  |  | | |  | |  | | |  | |  | | |  | |  | | |  | | |  | |  | | |  | |  | | |  | |
| F/F | 0.027 | a | 1.181 | a | | 0.955 | | a | | 0.171 | 1.406 | | | a | | | 2.883 | | a | | | 2.584 | | a | | | 0.197 | | 0.249 | | | a | | | 2.320 | | a | | | 2.003 | | a | | | 0.221 |
| F/G | 0.205 | ab | 1.540 | a | | 1.268 | | ab | | 0.171 | 2.556 | | | b | | |  | |  | | | 3.957 | | b | | | 0.197 | | 0.758 | | | ab | | | 2.915 | | a | | | 3.005 | | b | | | 0.221 |
| G/G | 0.680 | b | 1.541 | a | | 1.592 | | b | | 0.171 | 2.183 | | | b | | | 3.458 | | a | | | 3.375 | | b | | | 0.197 | | 1.327 | | | b | | | 2.910 | | a | | | 3.319 | | b | | | 0.221 |
| Large (Kg/plant) | | | | |  | |  | |  | |  |  |  | | |  | |  | | |  | |  | | |  | |  | | |  | | |  | |  | | |  | |  | | |  | |
| F/F | 0.051 | a | 0.637 | b | | 0.406 | | a | | 0.052 | 1.771 | | | b | | | 2.004 | | b | | | 1.865 | | b | | | 0.144 | | 0.427 | | | a | | | 1.624 | | b | | | 1.180 | | b | | | 0.145 |
| F/G | 0.284 | b | 0.712 | b | | 0.282 | | a | | 0.052 | 1.946 | | | b | | |  | |  | | | 1.677 | | b | | | 0.144 | | 0.775 | | | a | | | 1.639 | | b | | | 0.765 | | ab | | | 0.145 |
| G/G | 0.299 | b | 0.316 | a | | 0.253 | | a | | 0.052 | 1.208 | | | a | | | 1.139 | | a | | | 0.643 | | a | | | 0.144 | | 0.818 | | | a | | | 1.096 | | a | | | 0.673 | | a | | | 0.145 |
| Culls (% of total) | | | | |  | |  | |  | |  |  |  | | |  | |  | | |  | |  | | |  | |  | | |  | | |  | |  | | |  | |  | | |  | |
| F/F | 55.28 | b | 22.55 | a | | 20.60 | | a | | 3.94 | 24.67 | | | a | | | 13.44 | | a | | | 17.41 | | a | | | 3.03 | | 18.27 | | | a | | | 9.57 | | a | | | 18.12 | | ab | | | 2.71 |
| F/G | 47.69 | ab | 19.74 | a | | 24.79 | | a | | 3.94 | 20.41 | | | a | | |  | |  | | | 18.49 | | a | | | 3.03 | | 12.26 | | | a | | | 10.66 | | a | | | 10.82 | | a | | | 2.71 |
| G/G | 41.14 | a | 30.40 | a | | 27.89 | | a | | 3.94 | 35.11 | | | b | | | 20.99 | | a | | | 35.82 | | b | | | 3.03 | | 39.89 | | | b | | | 25.63 | | b | | | 21.62 | | b | | | 2.71 |
| Weather check (% of total) * | | | | | | |  | |  | |  |  |  | | |  | |  | | |  | |  | | |  | |  | | |  | | |  | |  | | |  | |  | | |  | |
| F/F | 2.45 | a | 1.38 | a | | 3.24 | | a | | 1.82 | 1.49 | | | a | | | 1.72 | | a | | | 0.69 | | a | | | 1.18 | | 1.00 | | | a | | | 2.09 | | a | | | 3.98 | | ab | | | 1.66 |
| F/G | 2.00 | a | 6.31 | a | | 1.00 | | a | | 1.82 | 1.07 | | | a | | |  | |  | | | 0.22 | | a | | | 1.18 | | 2.69 | | | a | | | 3.31 | | a | | | 1.45 | | a | | | 1.66 |
| G/G | 30.90 | b | 6.76 | a | | 2.82 | | a | | 1.82 | 9.84 | | | b | | | 3.87 | | a | | | 6.36 | | b | | | 1.18 | | 23.44 | | | b | | | 5.37 | | a | | | 7.24 | | b | | | 1.66 |
| Radial cracking (% of total) | | | | | | | | | | |  |  |  | | |  | |  | | |  | |  | | |  | |  | | |  | | |  | |  | | |  | |  | | |  | |
| F/F | 38.14 | a | 20.62 | a | | 14.77 | | a | | 3.34 | 14.26 | | | b | | | 8.30 | | a | | | 4.16 | | a | | | 1.28 | | 15.82 | | | a | | | 7.15 | | a | | | 15.41 | | a | | | 2.44 |
| F/G | 31.52 | a | 16.54 | a | | 18.55 | | a | | 3.34 | 8.43 | | | a | | |  | |  | | | 7.33 | | ab | | | 1.28 | | 7.28 | | | a | | | 7.52 | | a | | | 7.97 | | a | | | 2.44 |
| G/G | 27.17 | a | 23.56 | a | | 13.52 | | a | | 3.34 | 18.54 | | | b | | | 12.33 | | a | | | 9.99 | | b | | | 1.28 | | 31.85 | | | b | | | 23.08 | | b | | | 14.92 | | a | | | 2.44 |

* In fall 2017 and Fall 2018, analyses and mean separations were done based on log of percentage to meet model assumptions. Data shown here are displayed as percentages for comparison purposes. SE is the standard error of the mean. Within each background and season, different letters represent significantly different means (Tukey HSD test, alpha=0.05; based on a 4-replicate RCBD, [N=4]). The response value of each experimental unit comes from the plant yield per season averaged from 8 plants or percentage of total yield per season.

Table S8. Plant biomass and fruit pedicel length and width.

|  | Biomass (Kg) | | | | Pedicel length (mm) | | | | Pedicel width (mm) | | | |
| --- | --- | --- | --- | --- | --- | --- | --- | --- | --- | --- | --- | --- |
|  | Mean | SE* | Group | Mean | | SE | Group | Mean | | SE | Group |  |
| Fla. 7776 |  |  |  |  | |  |  |  | |  |  |  |
| F/F | 2.238 | 0.250 | b | 9.38 | | 0.24 | a | 4.54 | | 0.05 | c |  |
| F/G | 2.110 | 0.250 | b | 10.64 | | 0.24 | b | 4.33 | | 0.05 | b |  |
| G/G | 1.762 | 0.250 | a | 19.35 | | 0.24 | c | 3.31 | | 0.05 | a |  |
| Fla. 8022 |  |  |  |  | |  |  |  | |  |  |  |
| F/F | 1.993 | 0.250 | a | 11.37 | | 0.24 | a | 4.13 | | 0.05 | b |  |
| F/G | 1.974 | 0.257 | a | 12.37 | | 0.28 | b | 4.03 | | 0.06 | b |  |
| G/G | 1.777 | 0.250 | a | 18.47 | | 0.24 | c | 3.37 | | 0.05 | a |  |
| Fla. 8735 |  |  |  |  | |  |  |  | |  |  |  |
| F/F | 1.931 | 0.250 | b | 10.91 | | 0.24 | a | 3.99 | | 0.05 | b |  |
| F/G | 1.860 | 0.250 | ab | 12.10 | | 0.24 | b | 4.13 | | 0.05 | b |  |
| G/G | 1.619 | 0.250 | a | 19.19 | | 0.24 | c | 3.39 | | 0.05 | a |  |

SE is the standard error of the mean. Within each background, different letters represent significantly different means (Tukey HSD test, alpha=0.05, based on a 4-replicate RCBD, trialed three seasons [N=12]). The response value of each experimental unit comes from averaging the total weight of eight plants (plant biomass), or the average of 15 pedicels (three pedicels from each of five plants, pedicel length and width)

## Supplementary figures


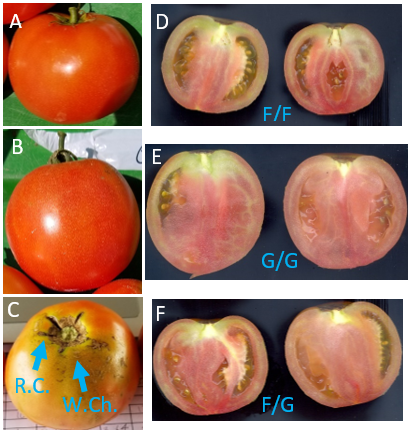


Figure S1. Typical fruit from plants with the genotypes: homozygous flat (A and D), homozygous globe (B and E), and heterozygous (F); and typical disorders in the cuticle of large-fruited fresh market tomatoes, radial cracking (R.C.) and weather check (W.Ch.) (C).


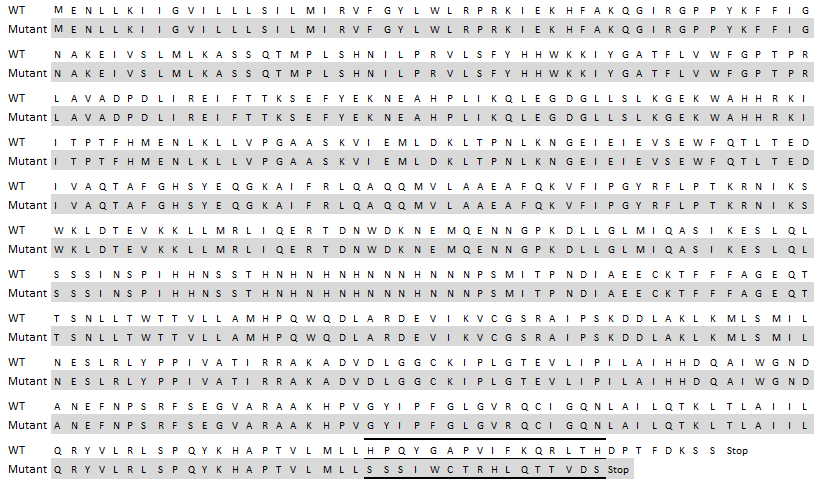


Figure S2. Predicted protein sequences of the wildtype and mutant brassinosteroid hydroxylase gene (Solyc12g006860). The single base pair deletion causes a frameshift mutation in the last exon of the gene, resulting in 16 amino acid mismatches (shown between the horizontal bars) and a premature stop codon that truncates the protein by eight amino acids.


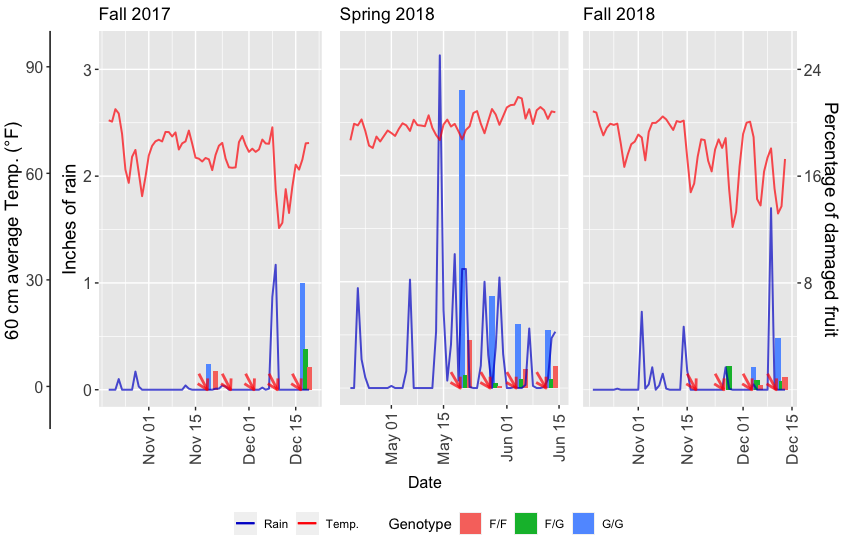


Figure S3. Weather check incidence in fruit of different genotypes at the *globe* locus and its relationship with rain. The blue line represents the total amount of rain for a given date. The red line represents the average daily temperature at 60 cm above the ground. The bars represent the percentage of fruit with weather check for a given harvest grouped by genotype (F/F, homozygous flat; F/G, heterozygous; G/G, homozygous globe). The red arrows point to the dates of harvest on each season. Weather data is shown for 1 month prior to the first harvest up to the end of the season (Source: Florida Automated Weather Network [FAWN]).


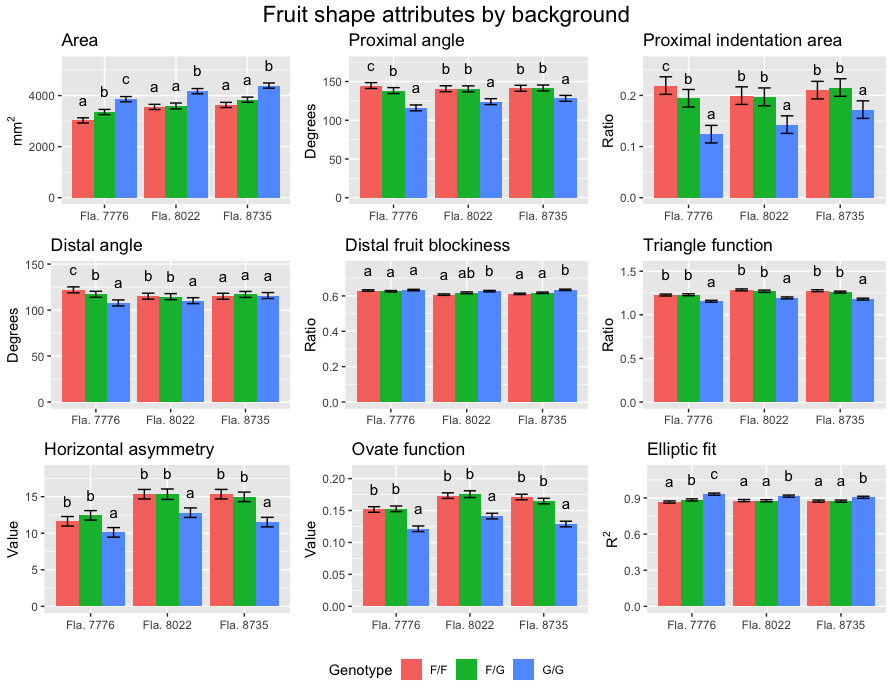


Figure S4. Mean values for nine fruit shape attributes measured across three seasons of trialing for each of three backgrounds and presented according to the genotype at the *globe* locus (F/F, homozygous flat; F/G, heterozygous; G/G, homozygous globe). The bars represent the standard error of the mean. Within each background, different letters represent significantly different means (Tukey HSD test, alpha=0.05, based on a 4-replicate RCBD, trialed three seasons [N=12]). The response value of each experimental unit comes from the average values of 24 fruits per experimental unit from second to fourth harvest.
